# Unexpected estradiol decline during ovarian stimulation monitoring affects cumulative live birth

**DOI:** 10.3389/fendo.2025.1658236

**Published:** 2025-10-20

**Authors:** Haixiao Chen, Kezhen Huang, Caihui Ma, Jie Geng, Lanlan Liu, Zhenfang Liu, Xuhong Na, Xiaoming Jiang, Jiali Cai, Jianzhi Ren

**Affiliations:** ^1^ Reproductive Medicine Center, Xiamen University Affiliated Chenggong Hospital, Xiamen, Fujian, China; ^2^ School of Medicine, Xiamen University, Xiamen, Fujian, China

**Keywords:** assisted reproductive biotechnologies (ART), ovarian stimulation, estradiol decline, follicular development, cumulative live birth rate

## Abstract

**Background:**

E_2_ is important in follicular development. During monitoring of stimulated cycles, serum levels of E_2_ are expected to increase steadily with follicle growth until final maturation. Unexpected E_2_ decline before triggering is reported in monitored COS cycles, yet its clinical significance remains controversial.

**Methods:**

The retrospective study was carried out in 27,487 conventional COS cycles at Xiamen University Affiliated Chenggong Hospital between January 2013 and December 2021. The occurrence of E_2_ decline during the monitoring was defined as the observation of a lower E_2_ value than the previous visit. Propensity matching and multivariate generalized linear models were used to analyze the association between E_2_ decline and cumulative live birth rates (CLBRs).

**Results:**

A total of 2,863 (10.3%) patients with E_2_ decline during COS monitoring were identified. In both unmatched and matched cohorts, the CLBRs were significantly decreased (unmatched cohort: 66.3% versus 55%, P<0.001, adjusted OR 0.83, 95% CI: 0.76,0.91; matched cohort: 59% versus 55%, P = 0.003, adjusted OR 0.84, 95%CI: 0.75,0.94). The E_2_ decline also decreased the oocyte yield and embryo yield, but the live birth following fresh transfer was not affected after matching. Mediation analyses showed that the decrease in CLBR was primarily due to decreased embryo yield in both unmatched (76.5% mediated, P = 0.002) and matched cohorts (72.5% mediated, P = 0.01). Subgroup analyses suggested that increasing the gonadotropin (Gn) dose did not improve CLBR (adjusted OR 0.91, 95% CI: 0.71,1.16). However, the patients with two consecutive declines in two visits may have worse outcomes (adjusted OR 0.72, 95% CI: 0.56,0.94).

**Conclusions:**

Although E_2_ is frequently monitored during COS, the value of routine E_2_ monitoring during COS has already been questioned. Our data suggest that the decline in E_2_ during COS monitoring is associated with the CLBR following a complete cycle, indicating it remains a critical biomarker in predicting the outcomes during COS. However, the overall size of the association is modest, and further attention should be paid to specific subgroups of patients, such as patients with consecutive E_2_ decline.

## Introduction

Controlled ovarian stimulation (COS) is a fundamental component of assisted reproductive technologies (ART) (1). It maximizes the potential for successful outcomes by stimulating multiple follicles to mature simultaneously, thereby increasing the availability of viable embryos for transfer. However, multiple follicle growth also raises concerns regarding the excessive ovarian response. During the process, follicular development and serum E_2_ are closely monitored to justify the decision to trigger oocyte maturation and in the prevention of ovarian hyperstimulation syndrome (OHSS) (2, 3).

The importance of E_2_ in follicular development in both natural and stimulated cycles has been well-established, as it is secreted by the granulosa cells (GC) in response to endogenous or exogenous follicle-stimulating hormone (FSH) stimulation to support the follicular growth and maturation (4). During monitoring of stimulated cycles, serum levels of E_2_ are expected to increase steadily with follicle growth until final maturation (5). However, unexpected E_2_ decline during COS monitoring is also reported in monitored COS cycles, yet its clinical significance remains controversial. An early study suggested that a decline in serum E_2_ before triggering is associated with a dramatically decreased pregnancy rate (6). However, milder effects or no effect of E_2_ decline during COS monitoring are also reported in other studies (7). The conflicting conclusions surrounding the clinical significance of E_2_ decline during COS can be attributed to various factors, such as limited sample size, heterogeneity of patients, or failure to adjust for important confounders. Importantly, previous research has predominantly focused on the outcomes of the fresh transfer, where patients with excessive ovarian response and patients with very poor response may both be excluded from the fresh transfer cycle. Selection bias may occur when paitents being included in the study basing of their exposure or outcomes status (8). In addition, the role of chance may also be a consideration when only fresh transfer is evaluated. Due to the morphology-based embryo selection having only limited discriminatory power (9), the “correct” embryos may be selected following multiple transfers.

Evaluating the cumulative birth rates taking into account all transfer attempts in a complete COS cycle (10) may minimize the bias associated with fresh transfer and the random effect of embryo selection. Moreover, the majority of the previous studies have associated the E_2_ decline with decreased fertilization or reduced embryo yield, suggesting fewer chances of transfer attempts (11–13). We hypothesize that an E_2_ decline observed during COS monitoring may compromise the cumulative live birth via the mediation of reduced embryo yield, even if the fresh transfer outcomes are not affected. The present study aims to evaluate the impact of E_2_ decline during COS monitoring on the cumulative live birth rate in a large COS cohort, exploring the mediation effect of embryo yield and the contribution of patient heterogeneity.

## Materials and methods

### Study subjects

We reviewed all patients who underwent ovarian stimulation for assisted reproductive technologies at Xiamen University Affiliated Chenggong Hospital between January 2013 and December 2021 for potential inclusion. The study was approved by the Institutional Review Board (IRB) of Xiamen University Affiliated Chenggong Hospital. Since the research was based on non-identifiable records, as approved by the IRB, obtaining informed consent was not required.

**Figure 1:**
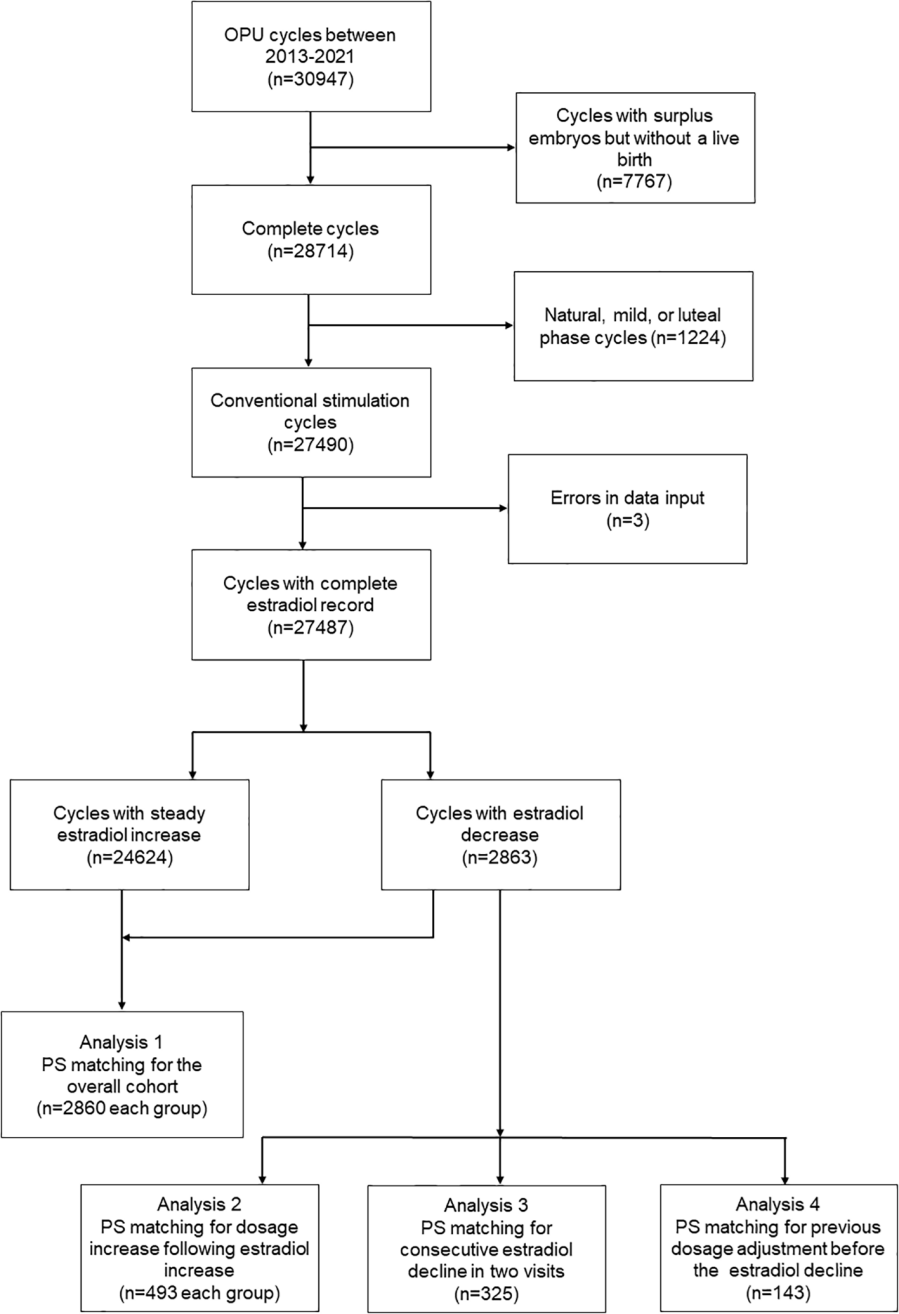
A flow chart for patient inclusion.

Due to the primary goal of the study being to evaluate the cumulative live birth following a complete cycle, the inclusion criteria were patients who achieved at least one live birth during the cycle or patients who had all their embryos transferred. The patients with surplus embryos but without a live birth (n=7767) were excluded. The exclusion criteria were patients who received non-conventional ovarian stimulation protocols such as natural, mild, or luteal phase cycles(n=1224), and cycles with errors in data input (n=3). The inclusion/exclusion criteria were detailed in a flowchart (**Figure 1**).

### Ovarian stimulation and monitoring

During the stimulation cycle, all patients received either the agonist or antagonist protocol and were administered follicle-stimulating hormone (FSH) or human menopausal gonadotropin (hMG), as previously described (14). The starting dose of gonadotropins (Gn) for ovarian stimulation ranged from 75 to 300 IU, determined according to the patient’s age, BMI, and ovarian reserve. Following the initiation of stimulation, the patient returned for the next visit in 4 to 6 days if the diameter of the follicle was less than 1.2 cm. When the diameter of the follicle was greater than 1.2 cm, the patient returned for monitoring every 1 to 2 days. In each visit during COS, the development of follicles was monitored under transvaginal ultrasonic examination, and the serum levels of serum follicle-stimulating hormone (FSH), estradiol (E2), and luteinizing hormone (LH) were also evaluated. Gn dosage adjustment may occur following a visit on the clinician’s decision, according to the outcomes of monitoring. The occurrence of a decrease in estradiol during the monitoring period was defined as observing an estradiol value lower than that of the previous follow-up.

Once ultrasonography confirmed that the average diameter of at least one follicle reached 18 mm or the diameter of two dominant follicles reached 17 millimeters, 200-250 μg of recombinant human chorionic gonadotropin injection (r-HCG, Ovitrelle, Merck Serono, Germany) would be administered subcutaneously to promote the final maturation of the follicles. Transvaginal ultrasound-guided oocyte retrieval was performed 35 to 37 hours after hCG administration. The occurrence of E_2_ decline during the monitoring was defined as the observation of a lower E_2_ value than the previous visit.

### Laboratory procedures

The oocytes were inseminated through either conventional *in vitro* fertilization (IVF) or intracytoplasmic sperm injection (ICSI) and cultured in individual droplets with oil overlay (OVOIL, Vitrolife, Göteborg, Sweden) in COOK culture mediums (COOK MEDICAL, Bloomington, IN). Conventional incubators (C200, Labotect, G¨ottingen, Germany) at 37 °C, 6% CO2, and 5% O2 in a humidified atmosphere were used for the *in vitro* culture. On day 3, the quality of embryos was scored manually according to the criteria of the Istanbul consensus (12). Patients would receive blastocyst culture according to the preference of the patients or clinicians. The blastocysts were scored according to the Gardner criteria (15).

For both cleavages and blastocysts, a vitrification protocol, employing 15% (v/v) dimethyl sulfoxide, 15% (v/v) ethylene glycol, and 0.6 M sucrose as cryoprotectants, was used for potential cryopreservation. A laser system (SATURN, RI, Falmouth, UK) was used for blastocyst collapse before vitrification.

### Pregnancy and live birth evaluation criteria

The criteria for judging live births in obstetrics follow the definition of the World Health Organization (WHO) (16). A live birth event is confirmed as a complete expulsion or extraction from its mother of a product of fertilization, irrespective of the duration of the pregnancy, which, after such separation, breathes or shows any other evidence of life, such as heart beat, umbilical cord pulsation, or definite movement of voluntary muscles, irrespective of whether the umbilical cord has been cut or the placenta is attached.

The cumulative live birth as the primary outcome of interest was defined as the first live birth event within a complete cycle. A complete cycle was defined as an OPU cycle that achieves at least one live birth event or has all resulting embryos transferred.

### Statistics

The association between E_2_ decline during ovarian stimulation monitoring and cumulative live birth was evaluated using a generalized linear model (GLM) and propensity score matching (PS-matching). For PS-matching, a MatchIt package in R software was used (17). The cobalt package (18) was used to test the balance. Standard differences (D) were calculated to evaluate the balance of the distribution of the baseline characteristics between the groups before and after PS matching. D < 0.1 was used as the threshold to indicate a negligible difference in the mean or prevalence of a covariate (19). The balance of covariates was also examined by the distribution of propensity score (distance) between matched groups.

The covariates and confounders for the analyses were selected based on previous knowledge and clinical experience with the assistance of a direct acyclic graph (DAG). The DAG was created by DAGitty software (https://dagitty.net/dags.html) and shown as a supplementary figure (**Supplementary Figure S1**). With a hypothesized association between E_2_ decline and cumulative pregnancy, the covariates that are associated with both the E_2_ decline (P<0.1 in the dataset) and cumulative live birth (20) were adjusted. These covariates included female age and BMI, fertility-related diagnosis (duration of infertility, tubal factor, endometriosis, PCOS), ovarian reserve markers (basal FSH, LH, and AFC), and ovarian stimulation (protocol and starting dosage). The analyses were also adjusted for potential confounders, including male factors (male age, BMI, total motile sperm count, and sperm normal morphology), insemination protocols (ICSI versus IVF), and clinical decisions (freeze-all and blastocyst culture) (20–23).

To test the hypothesis that E_2_ decline during monitoring affects the cumulative live birth via decreasing the embryo viability, we used the “mediation” packages to calculate the proportion of the total effect of E_2_ decline mediated by the average causal mediation effect (ACME) of the associated decreased embryo number.

To investigate whether a dose-dependent association exists between E_2_ decline and cumulative live birth, the association was also analyzed using generalized additive models (GAM) adjusted for the aforementioned covariates. The E_2_ decline was analyzed as continuous values and natural log-transformed. A “gratia” (Graceful ‘ggplot’-based graphics and utility functions for working with GAMs fitted using the ‘mgcv’) package was used to identify the potential turning point incorporated with the shape of the resulting curves. The package divided the range of E_2_ decline to 1000 points, and derivatives were calculated at each point based on the GAM model. Where the derivatives changed significantly (from indistinguishable from 0 to distinguishable from 0 or vice versa), the threshold was defined.

To explore the heterogeneity among patients with E_2_ decline, we also carried out PS-matching in patients with and without Gonadotropin (Gn) increase following E_2_ decrease, patients with and without consecutive decline at two visits, and patients with and without Gn adjustment before E_2_ decline.

E-values were introduced to measure the minimum strength of association that an unmeasured confounder would need to have to fully explain away the association of interest. The E-values were calculated using the R package “EValue”.

For descriptive analyses, continuous variables were analyzed using the Wilcoxon test, and categorical variables were analyzed using the chi-square test or Fisher’s exact test; P < 0.05 was considered to be significant. All analyses were performed using R statistical software 4.12 (24).

## Results

In this study, 30947 ART cycles were reviewed for potential inclusion, and 27487 cycles were finally included. The number of cycles that underwent at least one E_2_ decline was 2863 (10.3%). The characteristics of the patients are shown in **Table 1**. The patients encountering E_2_ decline during ovarian stimulation monitoring were associated with older female age, higher BMI, poorer AFC, and a lower proportion of agonist cycle. However, they also have a higher proportion of PCOS diagnoses and a history of delivering live births. Some of the characteristics of male counterparts, including age and Total motile sperm count (TMC), were also significantly different between patients with and without E_2_ decline. Following PS-matching, the standardized differences (D) for all the covariates were lower than 0.1, and the distribution of propensity scores (distance) was identical between comparison groups (**Supplementary Figure S2**).

**Table 1:** Characteristics of patients with and without unexpected estradiol decrease during monitoring.

Variables	Unmatched		*D	Matched		*D
	Non decreased	Decreased		Non decreased	Decreased	
	(N = 24624)	(N = 2863)		(N = 2860)	(N = 2860)	
Female age, years						
Mean(SD)	31.6(4.48)	32.6(4.85)	0.2016	32.4(4.84)	32.6(4.85)	0.0322
Median[Q1,Q3]	31.0[28.0,34.0]	32.0[29.0,36.0]		32.0[29.0,36.0]	32.0[29.0,36.0]	
BMI, kg/m^2^						
Mean(SD)	21.1(2.16)	21.5(2.20)	0.1697	21.5(2.08)	21.5(2.20)	0.009
Median[Q1,Q3]	21.2[19.5,22.8]	21.6[19.9,23.2]		21.6[20.0,23.0]	21.6[19.9,23.2]	
History of live birth	4658 (18.9%)	624 (21.8%)	0.0288	631 (22.1%)	624 (21.8%)	-0.0024
Duration of infertility, years						
Mean(SD)	4.23(3.08)	4.37(3.29)	0.0442	4.42(3.34)	4.38(3.29)	-0.014
Median[Q1,Q3]	3.50[2.00,5.60]	3.70[2.00,6.00]		3.80[2.00,6.00]	3.70[2.00,6.00]	
Tubal factor	15618 (63.4%)	1879 (65.6%)	0.022	1848 (64.6%)	1876 (65.6%)	0.0098
Polycystic ovarian syndrome	1417 (5.8%)	321 (11.2%)	0.0546	341 (11.9%)	320 (11.2%)	-0.0073
Endometriosis	2648 (10.8%)	267 (9.3%)	-0.0143	246 (8.6%)	267 (9.3%)	0.0073
Basal FSH, mIU/mL						
Mean(SD)	7.84(35.3)	10.4(103)	0.0252	8.19(20.4)	8.56(22.6)	0.0036
Median[Q1,Q3]	7.08[5.99,8.47]	7.39[6.15,9.29]		7.16[6.01,8.78]	7.39[6.15,9.29]	
Basal LH, mIU/mL						
Mean(SD)	4.90(2.93)	5.15(3.52)	0.0703	5.21(3.47)	5.15(3.52)	-0.0183
Median[Q1,Q3]	4.30[3.22,5.75]	4.34[3.19,5.95]		4.36[3.20,5.93]	4.34[3.19,5.95]	
AFC						
Mean(SD)	10.6(5.40)	10.3(6.54)	-0.0355	10.4(6.20)	10.3(6.51)	-0.0181
Median[Q1,Q3]	10.0[7.00,14.0]	9.00[5.00,14.0]		9.00[6.00,14.0]	9.00[5.00,14.0]	
Agonist protocol	20181 (82.0%)	1666 (58.2%)	-0.2377	1665 (58.2%)	1664 (58.2%)	-0.0003
Antagonist protocol	4443 (18.0%)	1197 (41.8%)		1195 (41.8%)	1196 (41.8%)	
Gn starting dose, IU						
Mean(SD)	197(39.4)	197(43.5)	-0.0133	196(41.2)	197(43.4)	0.0135
Median[Q1,Q3]	225[150,225]	225[150,225]		225[150,225]	225[150,225]	
IVF	17781 (72.2%)	2109 (73.7%)		2110 (73.8%)	2106 (73.6%)	
ICSI	6843 (27.8%)	754 (26.3%)	-0.0145	750 (26.2%)	754 (26.4%)	0.0014
Whole embryo blastocyst culture	6886 (28.0%)	607 (21.2%)	-0.0676	624 (21.8%)	607 (21.2%)	-0.0059
Freeze-all	5318 (21.6%)	510 (17.8%)	-0.0378	514 (18.0%)	509 (17.8%)	-0.0017
Male age, years						
Mean(SD)	33.3(5.12)	34.1(5.42)	0.1464	34.0(5.41)	34.1(5.42)	0.0195
Median[Q1,Q3]	33.0[30.0,36.0]	33.0[30.0,38.0]		33.0[30.0,37.0]	33.0[30.0,38.0]	
Male BMI, kg/m^2^						
Mean(SD)	23.9(3.35)	24.0(3.45)	0.0242	24.0(3.24)	24.0(3.44)	0.0121
Median[Q1,Q3]	23.7[21.5,26.0]	23.9[21.6,26.0]		23.9[21.7,26.0]	23.9[21.6,26.0]	
Sperm normal morphology, %						
Mean(SD)	7.04(4.85)	7.22(4.92)	0.0363	7.20(4.95)	7.22(4.93)	0.0035
Median[Q1,Q3]	6.00[4.00,9.00]	6.00[4.00,9.00]		6.00[4.00,9.00]	6.00[4.00,9.00]	
Total motile sperm count, 10^6^						
Mean(SD)	62.3(103)	73.3(476)	0.0232	63.5(69.3)	64.5(69.5)	0.0019
Median[Q1,Q3]	43.5[15.2,85.9]	44.8[17.2,88.3]		45.2[17.5,87.7]	44.7[17.2,88.2]	

Data were presented as mean ± SD and median [first quartile, third quartile] for continuous variables and n (percentage) for categorical variables. *D: Standardized difference. The absolute value of D is less than 0.1, cohorts can be considered to be balanced concerning the demographics being assessed.

**Table 2** shows the outcomes of patients with or without E_2_ decreased. The decrease in E_2_ levels during monitoring was associated with poorer ovarian response, which led to a prolonged duration of stimulation, increased total gonadotropin dosage, and a lower oocyte yield in both matched and unmatched cohorts. The number of mature oocytes, embryos, and high-quality embryos also decreased accordingly. These changes ultimately resulted in approximately a 4% difference (59% VS 55%, P = 0.002) in the cumulative live birth rate in the matched cohort. In the multivariate GLM analyses, the adjusted odds ratios (OR) for cumulative live birth were similar in the unmatched and the matched cohort (OR 0.83, 95% CI: 0.76,0.91 for the unmatched cohort; OR 0.84, 95%CI: 0.75,0.94). On the other hand, the live birth following fresh transfer did not significantly differ between patients with and without an E_2_ decline after PS-matching.

**Table 2:** Clinical outcomes of patients with and without unexpected estradiol decrease during monitoring.

Variables	Unmatched		P-value	Matched			P-value
	Non decreased	Decreased		Non decreased	Decreased		
	(N = 24624)	(N = 2863)		(N = 2860)	(N = 2860)		
Total dosage of Gn, IU							
Mean(SD)	2270(604)	2600(941)	<0.001	2080(596)	2600(941)		<0.001
Median[Q1,Q3]	2250[1800,2700]	2480[1960,3040]		2030[1650,2480]	2480[1950,3040]		
Gn situmilation duration, days							
Mean(SD)	11.3(2.49)	12.8(4.33)	<0.001	10.4(2.78)	12.8(4.33)		<0.001
Median[Q1,Q3]	11.0[10.0,13.0]	12.0[9.00,15.0]		10.0[8.00,12.0]	12.0[9.00,15.0]		
Estradiol on HCG day, pg/ml							
Mean(SD)	3310(2410)	2220(1870)	<0.001	2830(2170)	2220(1870)		<0.001
Median[Q1,Q3]	2820[1540,4470]	1610[886,3170]		2240[1210,3980]	1610[886,3170]		
Oocyte yield							
Mean(SD)	9.39(5.97)	7.14(5.46)	<0.001	8.20(5.78)	7.14(5.46)		<0.001
Median[Q1,Q3]	8.00[5.00,13.0]	6.00[3.00,10.0]		7.00[4.00,11.0]	6.00[3.00,10.0]		
Mature oocyte							
Mean(SD)	8.22(5.41)	6.35(5.03)	<0.001	7.18(5.24)	6.35(5.03)		<0.001
Median[Q1,Q3]	7.00[4.00,11.0]	5.00[3.00,9.00]		6.00[3.00,10.0]	5.00[3.00,9.00]		
Oocyte maturation rate, %							
Mean(SD)	87.7(17.3)	88.4(19.6)	<0.001	87.9(18.3)	88.4(19.6)		<0.001
Median[Q1,Q3]	93.3[80.0,100]	100[83.3,100]		100[80.0,100]	100[83.3,100]		
zygote							
Mean(SD)	7.28(4.98)	5.62(4.55)	<0.001	6.39(4.85)	5.62(4.56)		<0.001
Median[Q1,Q3]	6.00[4.00,10.0]	4.00[2.00,8.00]		5.00[3.00,9.00]	4.00[2.00,8.00]		
2 Pronuclei embryo							
Mean(SD)	5.81(4.13)	4.49(3.80)	<0.001	5.07(4.00)	4.49(3.80)		<0.001
Median[Q1,Q3]	5.00[3.00,8.00]	4.00[2.00,6.00]		4.00[2.00,7.00]	4.00[2.00,6.00]		
Cleavage							
Mean(SD)	6.24(4.38)	4.79(4.00)	<0.001	5.45(4.22)	4.79(4.00)		<0.001
Median[Q1,Q3]	5.00[3.00,9.00]	4.00[2.00,7.00]		4.00[2.00,8.00]	4.00[2.00,7.00]		
Cleavage 2 Pronuclei embryo							
Mean(SD)	5.70(4.07)	4.40(3.75)	<0.001	4.96(3.93)	4.41(3.75)		<0.001
Median[Q1,Q3]	5.00[3.00,8.00]	3.00[2.00,6.00]		4.00[2.00,7.00]	3.00[2.00,6.00]		
Good quality embryos							
Mean(SD)	3.51(3.10)	2.73(2.84)	<0.001	3.03(2.99)	2.73(2.84)		<0.001
Median[Q1,Q3]	3.00[1.00,5.00]	2.00[1.00,4.00]		2.00[1.00,4.00]	2.00[1.00,4.00]		
Cumulative live birth rate, %	16333 (66.3%)	1576 (55.0%)	<0.001	1688 (59.0%)	1574 (55.0%)		0.003
Fresh embryo transfer,N	(N = 18526)	(N = 2207)		(N = 2227)	(N = 2205)		
Fresh embryo transfer number							
1	6993 (37.7%)	912 (41.3%)	<0.001	857 (38.5%)		911 (41.3%)	0.131
2	11248 (60.7%)	1249 (56.6%)		1327 (59.6%)		1248 (56.6%)	
3	285 (1.5%)	46 (2.1%)		43 (1.9%)		46 (2.1%)	
Fresh blastocyst transfer	2673 (14.4%)	233 (10.6%)	<0.001	238 (10.7%)		233 (10.6%)	0.935
Fresh cycle live birth	9942 (53.7%)	1041 (47.2%)	<0.001	1051 (47.2%)		1039 (47.1%)	0.985
aOR (95% CI)*	ref	0.97(0.88,1.07)	0.525	ref		1.02(0.9,1.16)	0.745

All models were adjusted for female and male age, BMI, history of live birth, duration of infertility, tubal factor, PCOS, endometriosis, basic FSH, LH, antral follicle, agonist protocol, Antagonist protocol, Gn start dose, IVF, ICSI, whole embryo blastocyst culture, freeze-all, Sperm normal morphology, total mobil sperm count. The cumulative live birth rate was the dependent variable.

The mediation analyses suggested that the decreased cumulative live birth rate following E_2_ decline was largely mediated by the reduced number of mature oocytes or decreased embryo availability. The mature oocyte yield mediated 75.3% (P<0.001) of CLBR decline in the unmatched cohort and 78.3% (P = 0.006) in the matched cohort. Embryo number mediated 76.5% (P = 0.002) and 72.5% (P = 0.01) in the unmatched and matched cohorts, respectively.

**Figure 2:**
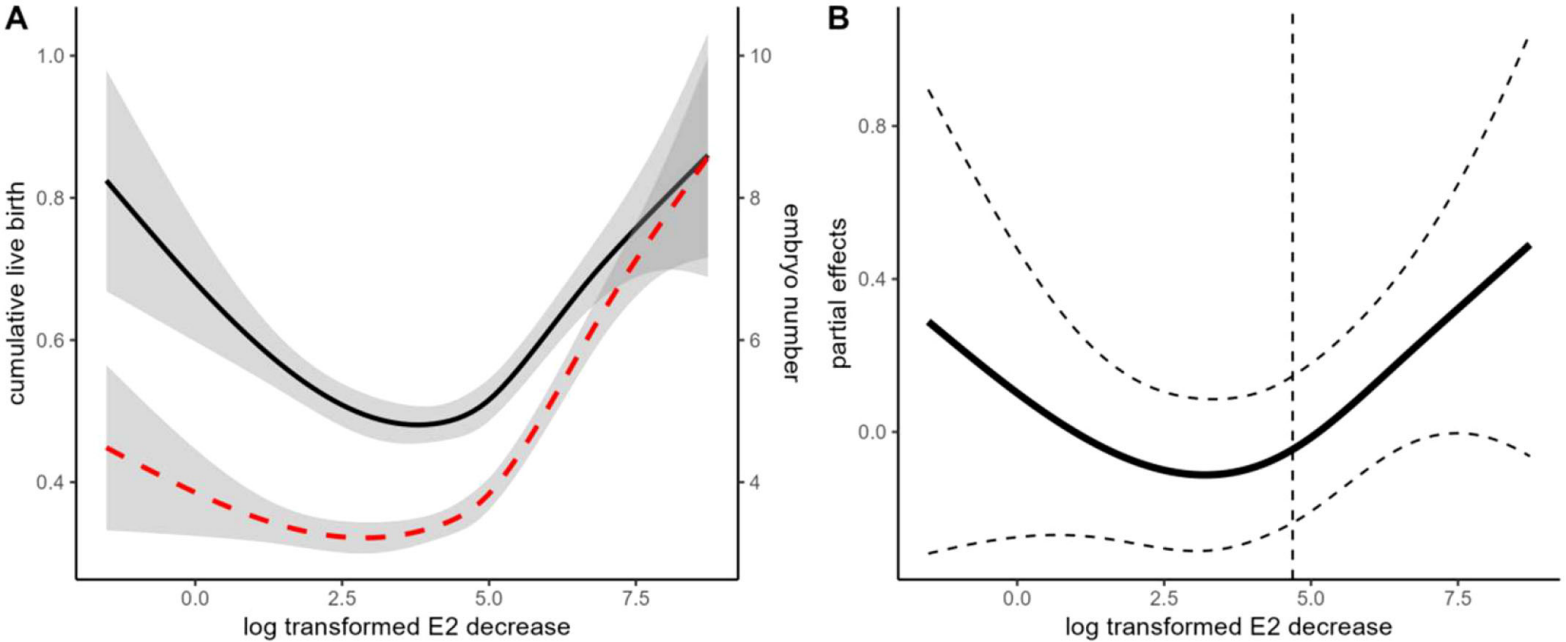
Association between the degree of E_2_ decline and cumulative live birth rate (CLBR). **(A)** Unadjusted GAM models indicate the association between E_2_ decline and CLBR and embryo yield. The black solid line indicates the association with CLBR. The Red dashed line indicates the association with embryo yield. **(B)** Adjusted GAM spline for E_2_ decline in association with CLBR. The dashed line indicates the inflection point according to the “gratia” package. The model is adjusted for female age and BMI, fertility-related diagnosis (duration of infertility, tubal factor, endometriosis, PCOS), ovarian reserve markers (basal FSH, LH, and AFC), and ovarian stimulation (protocol and starting dosage), male age, BMI, total motile sperm count, sperm normal morphology, insemination protocols (ICSI versus IVF), and clinical decisions (freeze-all and blastocyst culture).

The GAM model suggested a U-shaped association between E_2_ decline and cumulative live birth (edf=2, P = 0.01). The cumulative live birth rates were negatively associated with the degree of E_2_ decline when the differences between the two visits were less than 108.9 pg/ml (natural log-transformed 4.68) (**Figure 2**). However, the negative association diminished in cycles with greater E_2_ decline. The OR for the negative association was 0.862(95%CI: 0.765-0.972). When patients were stratified according to COS protocols, the pattern of association between E_2_ decline and CLBR was similar between the agonist and antagonist protocols (**Supplementary Figure S3**). However, the dose-response lacked statistical significance in patients with the antagonist protocol (p=0.38). We further visualized the difference between the dose-response curves of agonist and antagonist protocols in the GAM model. It shows that the CLBR was significantly lower in the antagonist protocol than the agonist protocol when the E_2_ decline is higher than 4.28 pg/ml (log transformed 1.455), and the difference further extends when the degree of E2 decline further increases. Considering a much lower CLBR in the antagonist protocol than that in the agonist protocol (37.3% versus 71.1%), the lack of association in the antagonist protocol would be due to a lack of power.

**Table 3** demonstrates the characteristics of the E_2_ decline during monitoring. Most of the E_2_ decline occurred between the 8th and 13^th^ day of stimulation, with a median E_2_ decline of 244 pg/ml. The E_2_ decline only had a limited association with follicle count and mean diameter at the visit. As shown in a Pearson correlation matrix (**Supplementary Figure S4**), the degree of E_2_ decline did not correlate with follicle diameter changes in comparison with the previous visit, and only a low correlation with follicle count changes. A modest correlation (r=0.181) between the degree of E_2_ decline and the Gn dosage change in the previous visit was also observed. However, only a small proportion of patients with E_2_ decline (5.1%, n=146) were associated with a Gn dosage decrease in the previous visit. On the other hand, 18.8% (n=538) of the patients underwent Gn dosage increase following the observation of decline. In addition, about 11.4% of the patients underwent consecutive E_2_ decline at two visits. Since the descriptive data suggested that there was considerable heterogeneity in patients with E_2_ decline, we further investigated the effects of heterogeneity on cumulative outcomes.

**Table 3:** Heterogeneity of patients with estradiol decline during monitoring.

Variables	(N = 2863)
The stimulation day the decline occurs	
Mean (SD)	10.6(3.71)
Median [Min, Max]	10.0[8.00,13.0]
Absolute E_2_ decline, pg/ml	
Mean (SD)	-244(463)
Median [Min, Max]	-82.0[-262,-20.0]
Percentage of E_2_ decline, %	
Mean (SD)	20.5(21.6)
Median [Min, Max]	12.8[5.26,27.5]
Consecutive decline in the next visit	325 (11.4%)
Dosage change following the decline, IU	
Mean (SD)	9.87(24.0)
Median [Min, Max]	0[0,0]
Follicular diameter changes at the decline	
Mean (SD)	1.81(2.23)
Median [Min, Max]	1.50[0.600,2.60]
Dosage used when the decline occurs, IU	
Mean (SD)	139(107)
Median [Min, Max]	188[0,225]
Follicle count changes at the decline	
Mean (SD)	-0.161(2.68)
Median [Min, Max]	0[-1.00,1.00]
Dosage adjustment before the decline, IU	
Mean (SD)	-0.0830(17.0)
Median [Min, Max]	0[0,0]
Dosage decrease before the decline (%)	146 (5.1%)

Because the Venn diagram (**Supplementary Figure S5**) suggests that there was limited overlapping between patients who underwent Gn dosage decrease before E_2_ decline, patients with Gn dosage increase following E_2_ decline, and patients with consecutive E_2_ decline in two consecutive visits, we analyzed them independently in multivariate models. In multivariate glm models, we compared the aforementioned E_2_ decline subgroups (patients with and without Gn increase following E_2_ decrease, patients with and without consecutive decline at two visits, and patients with and without Gn decrease before E_2_ decline) with patients without E_2_ decline (**Supplementary Figure S6**). The size of association (ORs) did not significantly differ between the patients with and without Gn increase following E_2_ decrease or patients with and without Gn decrease before E_2_ decline (**Supplementary Figure S6**). However, the OR in patients with two consecutive E_2_ declines (0.58, 95% CI: 0.45,0.74) was significantly lower than that in patients without (OR 0.87, 95%CI: 0.79,0.96).

To confirm the effect of heterogeneity, we also carried out PS-matching in the subgroups (detailed in **Supplementary Table S1**-**6**, **Supplementary Figures S7**-**9**), and the summarized results are shown in **Table 4**. Increasing the dosage of Gn following E_2_ decline appeared to improve cumulative live birth, but the difference diminished following matching or multivariate analyses. On the other hand, the patients with consecutive E_2_ decline in two visits tended to have lower cumulative live birth rates than patients without in both the unmatched and matched cohorts. Although the P values became marginal in the matched cohort due to reduced sample size, the size of the association was robust in both the unmatched and matched cohorts, with or without multivariate adjustment.

**Table 4:** The effect of heterogeneity in patients with estradiol decline during monitoring on cumulative live birth.

Subgroup	Status	N	Cumulative live birth (%)	Crude OR (95% CI)	aOR (95% CI)
Unmatched					
Dosage increase following estradiol increase	no	2325	1213 (52.2%)	ref	ref
	yes	538	363 (67.5%)	1.9(1.56,2.32)	0.91(0.71,1.16)
	P-value		<0.001	<0.001	0.425
Consecutive estradiol decline in two visits	no	2538	1416 (55.8%)	ref	ref
	yes	325	160 (49.2%)	0.77(0.61,0.97)	0.72(0.56,0.94)
	P-value		0.029	0.026	0.017
Previous dosage adjustment before estradiol decline	no	2717	1460 (53.7%)	ref	ref
	yes	146	116 (79.5%)	3.33(2.21,5.01)	1.67(1.07,2.6)
	P-value		<0.001	<0.001	0.025
Matched					
Dosage increase following estradiol increase	no	493	331 (67.1%)	ref	ref
	yes	493	331 (67.1%)	1(0.77,1.3)	0.94(0.66,1.34)
	P-value		>0.999	>0.999	0.999
Consecutive estradiol decline in two visits	no	325	183 (56.8%)	ref	ref
	yes	325	159 (49.4%)	0.74(0.54,1.01)	0.72(0.5,1.03)
	P-value		0.069	0.058	0.07
Previous dosage adjustment before estradiol decline	no	143	98 (68.5%)	ref	ref
	yes	143	114 (79.7%)	2.27(1.19,4.32)	0.95(0.45,2)
	P-value		0.043	0.012	0.901

All models were adjusted for female and male age, BMI, history of live birth, duration of infertility, tubal factor, PCOS, endometriosis, basic FSH, LH, antral follicle, agonist protocol, Antagonist protocol, Gn start dose, IVF, ICSI, whole embryo blastocyst culture, freeze-all, Sperm normal morphology, total mobil sperm count. The cumulative live birth rate was the dependent variable.

E-values of these associations (**Supplementary Table S8**) suggested that the minimal strength of the association that an unknown confounding needs to explain away the association between E_2_ decline and CLBR was 1.43(95%CI: 1.27,1.56), which was greater than that of the well-known predictor, female age (1.25, 95%CI: 1.23,1.27). For patients with a consecutive estradiol decline, the E-value for E_2_ decline was even higher (1.95, 95%CI: 1.6,2.35).

## Discussion

In our study, we observed a decline in E_2_ levels in 2,863 out of 27,487 patients undergoing COS, corresponding to an incidence of 10.63%. This incidence indicates that E_2_ decline is a moderate occurrence in the context of COS, highlighting the need for further investigation into its clinical significance. However, the data also shows a modest association between the E_2_ decline and decreased CLBR following complete cycles. Compared with propensity score-matched controls, a 4% difference in CLBR was observed. Therefore, E_2_ decline may be a potential detriment in COS treatment as a part of ART.

Historically, the impact of E_2_ decline on the ART outcomes is debatable and the majority of the studies focused on the fresh transfer cycles. Some studies reported a dramatic decrease in pregnancy rates following E_2_ decline. For instance, Kulshrestha, et al. reported a 14% pregnancy rate in 23 patients undergoing pre-triggering E_2_ decline (6). In a cohort of 78 patients with spontaneous E_2_ decline before hCG administration, Fisher et al. found that clinical pregnancy rates were significantly reduced (13% vs. 39%, p = 0.012) (11). In contrast, Styer et al (7) reported no significant differences in live birth or pregnancy loss rates between cycles with E_2_ decline and controls in GnRH agonist-downregulated protocols (n=65). Other studies demonstrated a modest decrease in pregnancy and live births following E_2_ decline (12, 25). This discrepancy may stem from variations in study populations or protocols. Many of the studies have only limited sample sizes (dozens of patients per group), which could be easily affected by random variation and thus give opposite conclusions (26). In addition, important determinators of embryo transfer outcomes, such as the number of embryos transferred and the stage of the transferred embryo, are adjusted in none of the above-mentioned studies. Our study contributes to the ongoing debate by adding evidence in a patient cohort with a large sample size and confounders adjusted, showing a neutral effect of E_2_ decline on fresh transfer.

Despite conflicting conclusions on the outcomes following the transfer, the majority of the previous studies agree that E_2_ decline during COS may decrease embryo yield for subsequent treatment (11–13). It also raises concerns regarding embryo developmental competence. In a donor-recipient cohort, Cobo et al’s study indicates that a fall of ≥30% in serum E2 concentration during ovarian stimulation in donors negatively affects pregnancy rates and embryo quality in recipients (27). The authors warn against a decreased embryo quality following E_2_ decline and suggest considering a cycle cancellation. Corroborating these studies, our study demonstrates that E_2_ decline significantly reduced the total number of oocytes, embryos, and high-quality embryos, mediating a decrease in CLBR following complete cycles. However, considering the limited effect size, we do not recommend cycle cancellation due to the E_2_ decline.

The degree of E_2_ decline is another potential concern. Previous studies may use arbitrary thresholds such as 10% or 30% to define E_2_ decline (7, 11–13, 25, 28). However, since E_2_ is secreted in granulosa cells, the magnitude of both absolute and relative E_2_ decline is largely dependent on the size of the patients’ growing follicle cohort. Excessive E_2_ decline could only be expected in hyperresponders. On the other hand, the marginal benefit of CLBR per embryo number increase would also be narrowed in hyperresponders (**Supplementary Figure S10**). Considering the decrease in CLBR was primarily mediated by embryo yield, it may explain the U-shaped curve observed in GAM analyses. Nevertheless, our dose-response curves still suggested that patients with a suboptimal response (eg. embryo <=4) are more vulnerable to the E_2_ decline, and an E_2_ decrease of around 100 pg/ml might be a threshold of concern.

We also explored the heterogeneity of patients encountering E_2_ decline. Several previous studies have distinguished between spontaneous E_2_ decline and intended E_2_ reductions via Gn dose adjustment (7, 13), showing that suboptimal outcomes are only found in patients with spontaneous E_2_ decline. Echoing the studies, we also found patients having a Gn adjustment prior to E_2_ decline tended to have an optimal outcome, though the small number of such patients hampered drawing a firm conclusion. On the other hand, we noted that a significant part of the patients underwent consecutive E_2_ decline in two visits, which led to a poorer prognosis. Although the mechanism is not known, the decline may reflect compromised granulosa cell function or accelerated follicular atresia in follicles (27). While COS recruits a cohort of antral follicles by administration of exogenous Gn, follicles in the cohort may have different degrees of atresia which is probably due to unsynchronized growth (29). The follicles destinated to atresia before triggering produce E_2_ in a gradually reduced production rate but resulted in no oocyte. The consecutive decline would suggest a high degree of atresia in the cohort and impaired follicular dynamics.

When encountering a decline in E_2_ levels during COS, clinicians may face management decisions, such as maintaining the scheduled dose of Gn or Gn dosage adjustment during COS. Increasing Gn dosage during COS is a commonly used strategy to maximize the ovarian response and oocyte yield (30, 31). However, its usefulness in patients with E_2_ decline is less clear. Styer et al. reported no significant differences in live birth rates in patients with E_2_ decline, regardless of whether the dose of gonadotropins was adjusted (7), suggesting increasing the Gn dosage following the occurrence of E_2_ decline may not be effective management. Our subgroup analyses also support the point in a larger, multivariate-adjusted cohort. Nevertheless, the evidence so far is based on retrospective studies, and further study is warranted to investigate to optimize the management of these patients.

## Strengths and limitations

The strengths of the study may include a larger sample size than previous studies (7, 11–13, 25, 28), with multivariate adjustment and reporting of CLBR following the complete cycle. By focusing on CLBR, our study bridges a gap in existing literature, statistically linking repeated E_2_ declines to diminished reproductive success.

The limitations of this study lie in its retrospective research design, which inherently carries the risk of biases related to the selection and evaluation of clinical cases. Although we employed multivariate analysis to adjust for confounding variables, there is still a possibility that unknown or unmeasured factors may have influenced the research results. For instance, although the culture system, equipment, and staff remained stable during the study period, unmeasured variations in the laboratory environment, such as fluctuation in air quality, may contribute to confounding. Nevertheless, the E-values suggest that the minimal strength of the association that an unknown confounding needs to explain away the association between E_2_ decline and CLBR was greater than the well-known predictor, female age. These limitations emphasize the need for caution when interpreting the research findings, and also highlight the importance of using prospective research methods in the future to validate our findings and further elucidate the relationship between E_2_ levels and reproductive outcomes during the COS process.

In addition, due to the study being observational, the study cohort was also involved in interventions such as Gn dose adjustment during COS. Such interventions are based on the clinicians` decisions on individual cases and inevitably introduce bias. Although we also carried out subgroup analyses, they may be underpowered and heterogeneous.

Finally, the reasons for the occurrence of continuous decreases in E_2_ are still unclear. It is possible that patients who underwent E_2_ decline for different reasons are misclassified in our study and previous ones, which resulted in a skewed conclusion.

## Conclusions

Although E_2_ is frequently monitored during COS (32), the value of routine E_2_ monitoring during COS has already been questioned (33). Our data suggest that the decline in E_2_ during COS monitoring is associated with the CLBR following a complete cycle, indicating it remains a critical biomarker in predicting the outcomes during COS. However, the overall size of the association is modest, with an E-value (1.43, 95%CI: 1.27,1.56) comparable to that of female age. Further attention should be paid to specific subgroups of patients, such as patients with consecutive E_2_ decline.

## Data Availability

The raw data supporting the conclusions of this article will be made available by the authors, without undue reservation.

## Supplementary material

Supplementary Figure 1 A directed acyclic graph for covariate selection.

Supplementary Figure 2 Association between the degree of E_2_ decline and cumulative live birth rate (CLBR) in antagonist and agonist protocols. **(A)** GAM models indicate the association between E_2_ decline and CLBR in antagonist and agonist protocols. The blue line indicates the association in the antagonist protocol. The bed line indicates the association in the agonist protocol. **(B)** The difference between GAM splines in antagonist and agonist protocols. The shade indicates 95% confidence intervals of the difference. The red area indicates. All models are adjusted for female age and BMI, fertility-related diagnosis (duration of infertility, tubal factor, endometriosis, PCOS), ovarian reserve markers (basal FSH, LH, and AFC), and ovarian stimulation (protocol and starting dosage), male age, BMI, total motile sperm count, sperm normal morphology, insemination protocols (ICSI versus IVF), and clinical decisions (freeze-all and blastocyst culture).

Supplementary Figure 3 The distribution of propensity score in patients matched for E_2_ decline. The green shades indicate patients with E_2_ decline and the pink shades indicate controls.

Supplementary Figure 4 A correlation matrix between E_2_ decline and changes in gonadotropin dosage and follicles. A blank grid indicates no significant association. FF_diameter_change, changes in mean diameters of the monitored follicles at the visit; Gn_change_previously, changes in Gn dosage in previous visit; E_2__decrease, the degree of E_2_ decline in comparison with the previous visit; Gn_dosing_change, changes in Gn dosage following the occurrence of E_2_ decline, FF_count_change, changes in the count of the monitored follicles at the visit.

Supplementary Figure 5 Overlapping of heterogeneous subgroups of patients with E_2_ decline. Set 1, patients with gonadotropin (Gn) increase following E_2_ decline, Set 2, patients with and without consecutive decline at two visits, Set 3, patients with and without Gn adjustment before E_2_ decline.

Supplementary Figure 6 Odds ratios (OR) comparing E_2_ decline subgroups with patients without E_2_ decline. All models are adjusted for female age and BMI, fertility-related diagnosis (duration of infertility, tubal factor, endometriosis, PCOS), ovarian reserve markers (basal FSH, LH, and AFC), and ovarian stimulation (protocol and starting dosage), male age, BMI, total motile sperm count, sperm normal morphology, insemination protocols (ICSI versus IVF), and clinical decisions (freeze-all and blastocyst culture).

Supplementary Figure 7 The distribution of propensity score in patients matched for gonadotropin (Gn) increase following E_2_ decline. The green shades indicate patients with Gn increase and the pink shades indicate control patients with E_2_ decline.

Supplementary Figure 8 The distribution of propensity score in patients matched for consecutive E_2_ decline at two visits. The green shades indicate patients with consecutive E_2_ decline and the pink shades indicate control patients with E_2_ decline.

Supplementary Figure 9 The distribution of propensity score in patients matched for gonadotropin (Gn) adjustment before E_2_ decline. The green shades indicate patients with Gn adjustment and the pink shades indicate control patients with E_2_ decline.

Supplementary Figure 10 The association between embryo yield and cumulative birth.
