# Supplementary figures and images for: Unexpected estradiol decline during ovarian stimulation monitoring affects cumulative live birth
Source: Front Endocrinol (Lausanne). 2025 Oct 20;16:1658236. doi: 10.3389/fendo.2025.1658236 (PMC12580126; doi:10.3389/fendo.2025.1658236)

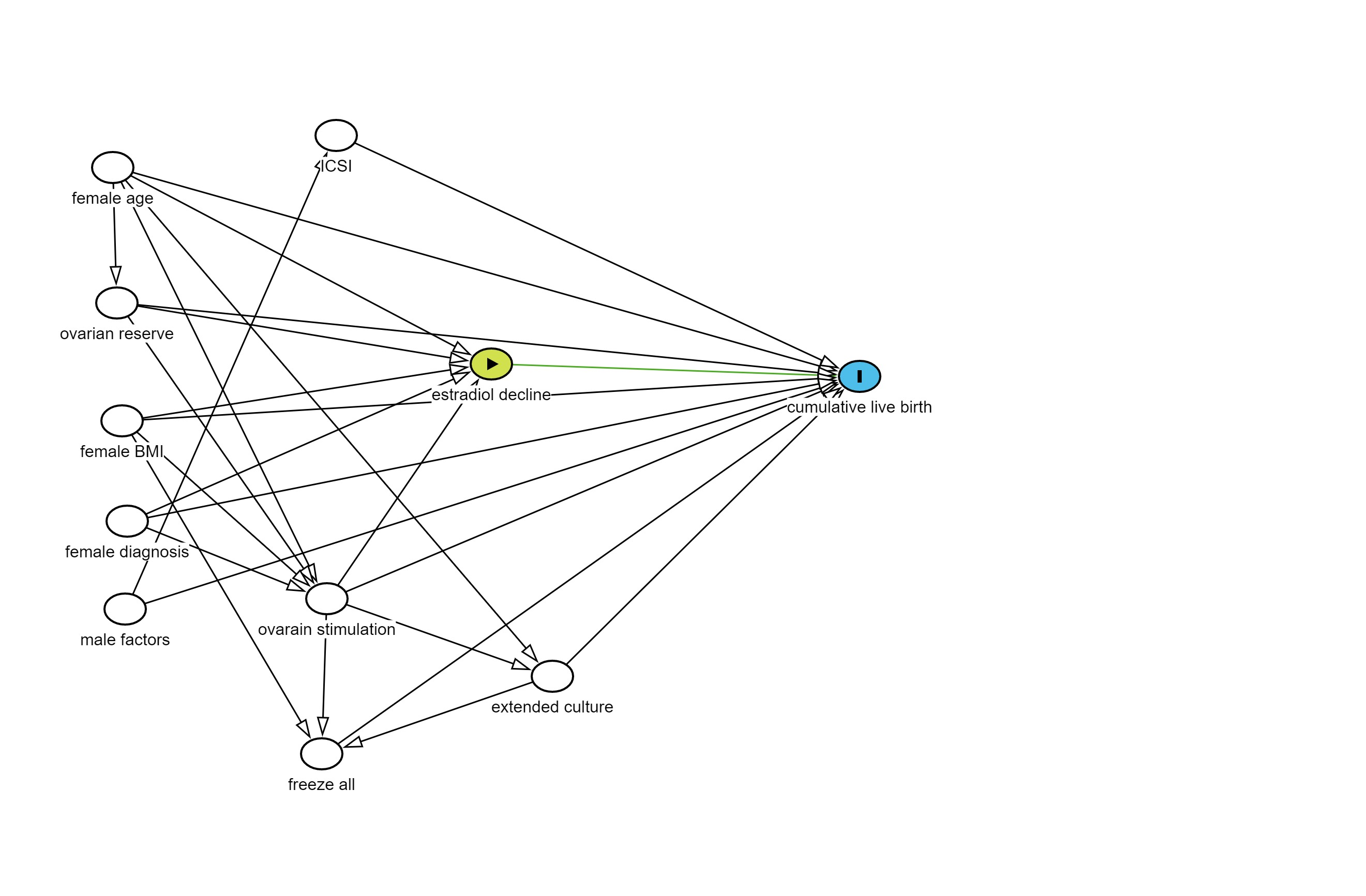

Supplement: Supplementary Figure 1 — A directed acyclic graph for covariate selection. [file Image1.jpeg]

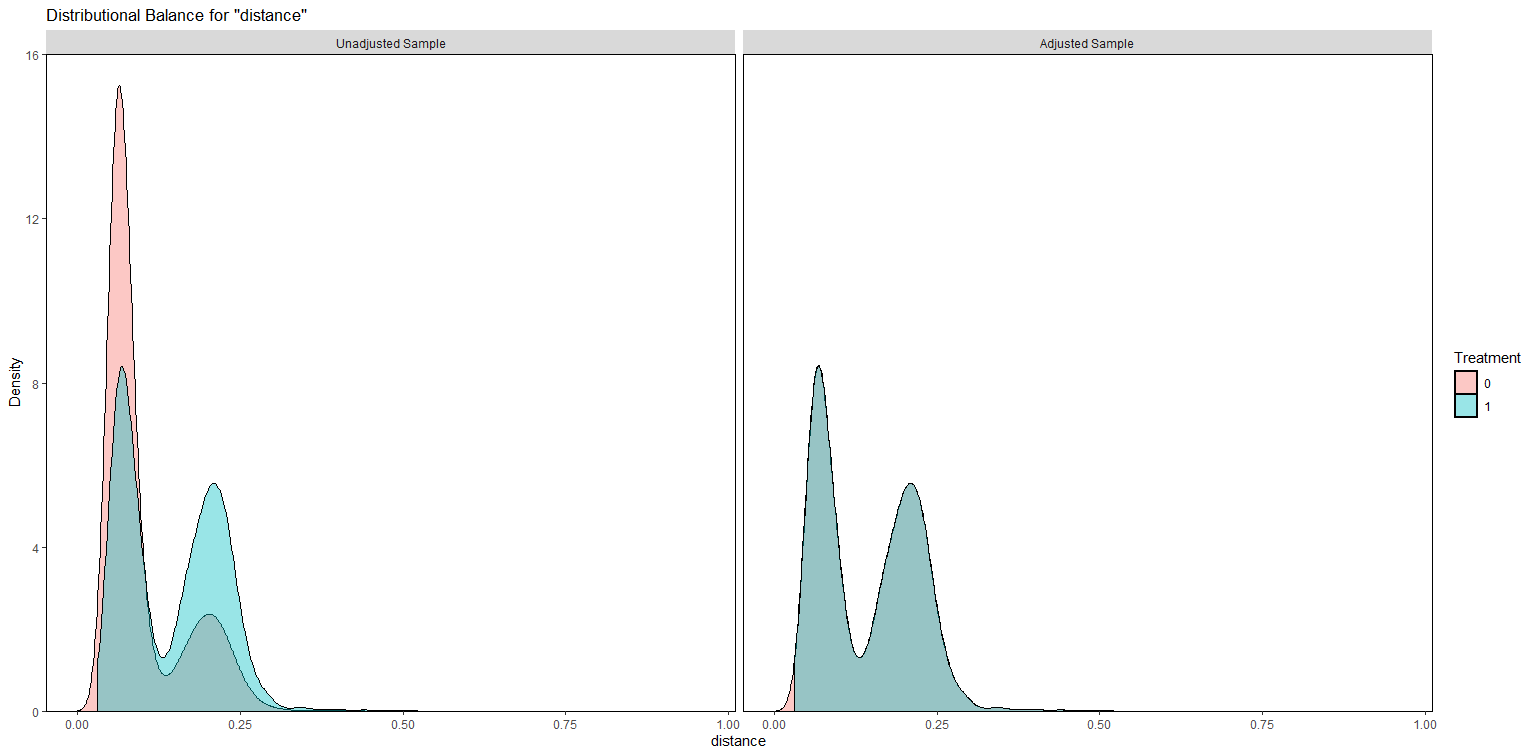

Supplement: Supplementary Figure 2 — Association between the degree of E2 decline and cumulative live birth rate (CLBR) in antagonist and agonist protocols. (A) GAM models indicate the association between E2 decline and CLBR in antagonist and agonist protocols. The blue line indicates the association in the antagonist protocol. The bed line indicates the association in the agonist protocol. (B) The difference between GAM splines in antagonist and agonist protocols. The shade indicates 95% confidence intervals of the difference. The red area indicates. All models are adjusted for female age and BMI, fertility-related diagnosis (duration of infertility, tubal factor, endometriosis, PCOS), ovarian reserve markers (basal FSH, LH, and AFC), and ovarian stimulation (protocol and starting dosage), male age, BMI, total motile sperm count, sperm normal morphology, insemination protocols (ICSI versus IVF), and clinical decisions (freeze-all and blastocyst culture). [file Image2.tiff]

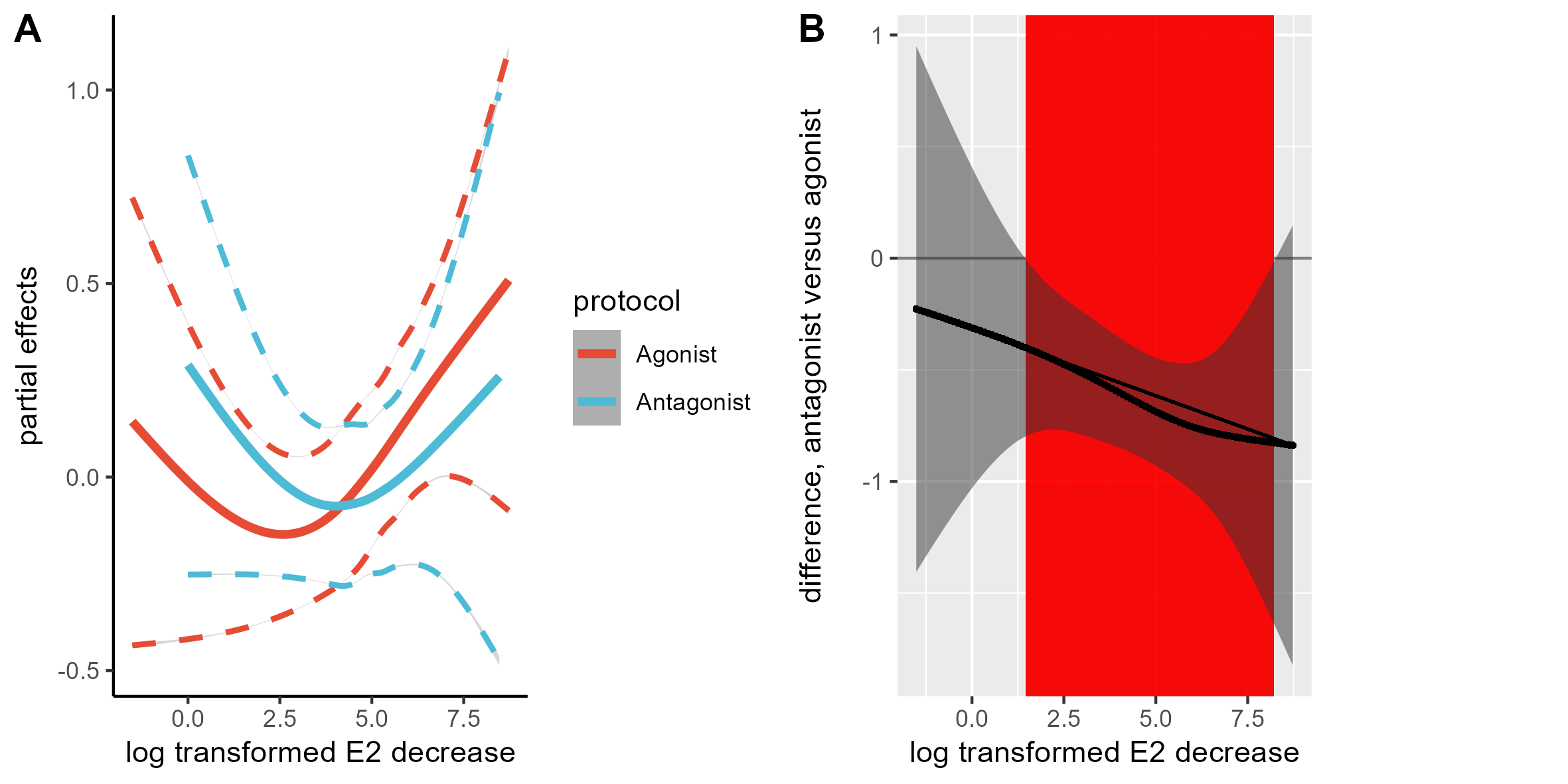

Supplement: Supplementary Figure 3 — The distribution of propensity score in patients matched for E2 decline. The green shades indicate patients with E2 decline and the pink shades indicate controls. [file Image3.tiff]

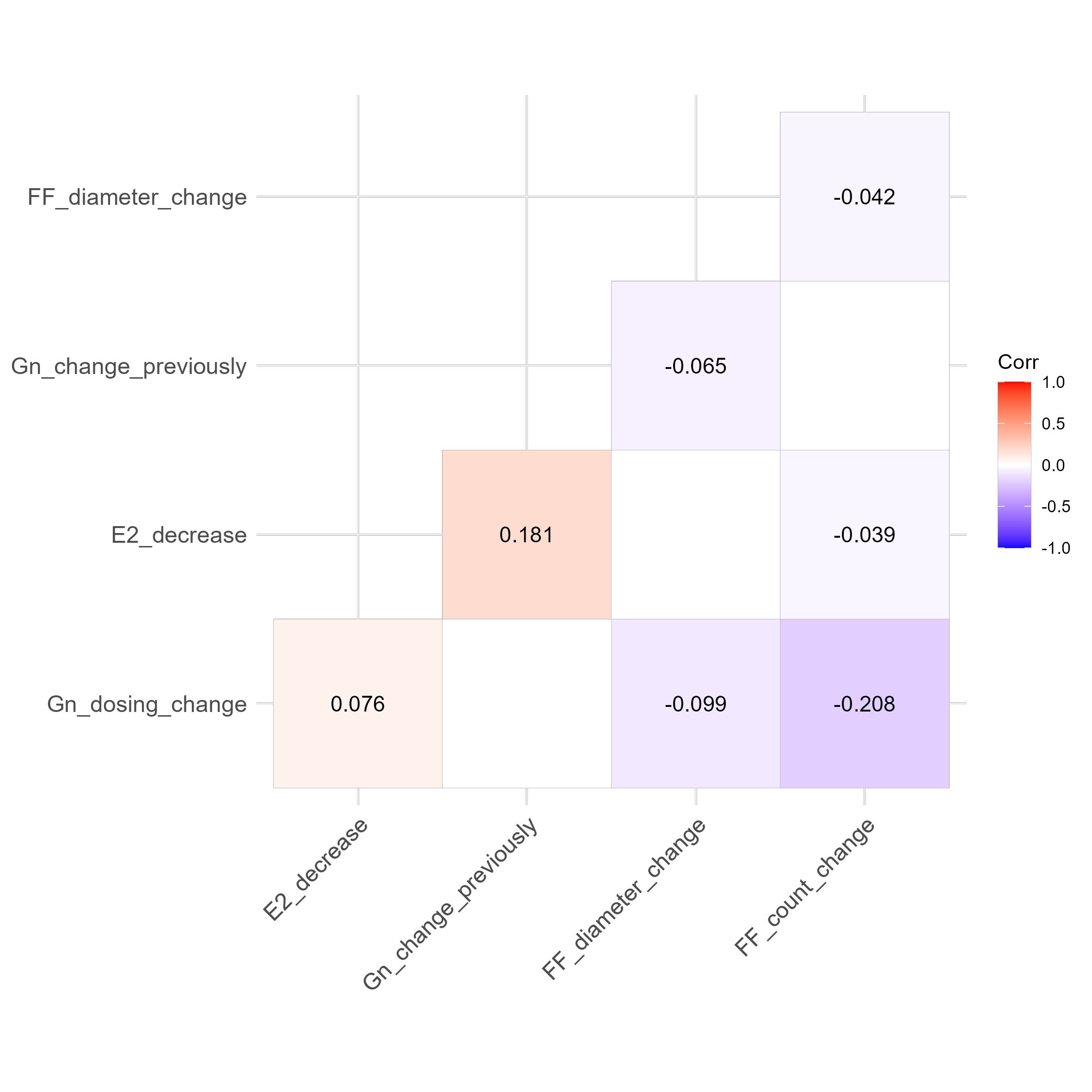

Supplement: Supplementary Figure 4 — A correlation matrix between E2 decline and changes in gonadotropin dosage and follicles. A blank grid indicates no significant association. FF_diameter_change, changes in mean diameters of the monitored follicles at the visit; Gn_change_previously, changes in Gn dosage in previous visit; E2_decrease, the degree of E2 decline in comparison with the previous visit; Gn_dosing_change, changes in Gn dosage following the occurrence of E2 decline, FF_count_change, changes in the count of the monitored follicles at the visit. [file Image4.tiff]

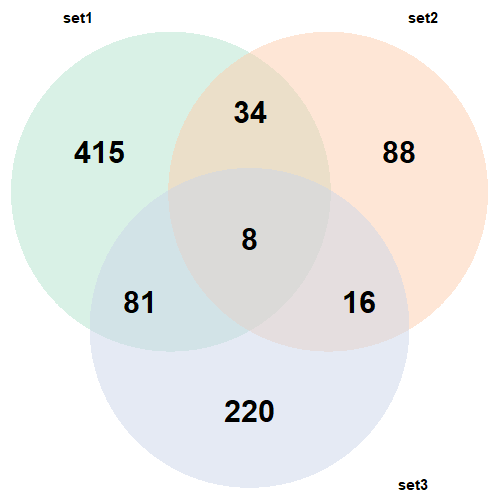

Supplement: Supplementary Figure 5 — Overlapping of heterogeneous subgroups of patients with E2 decline. Set 1, patients with gonadotropin (Gn) increase following E2 decline, Set 2, patients with and without consecutive decline at two visits, Set 3, patients with and without Gn adjustment before E2 decline. [file Image5.png]

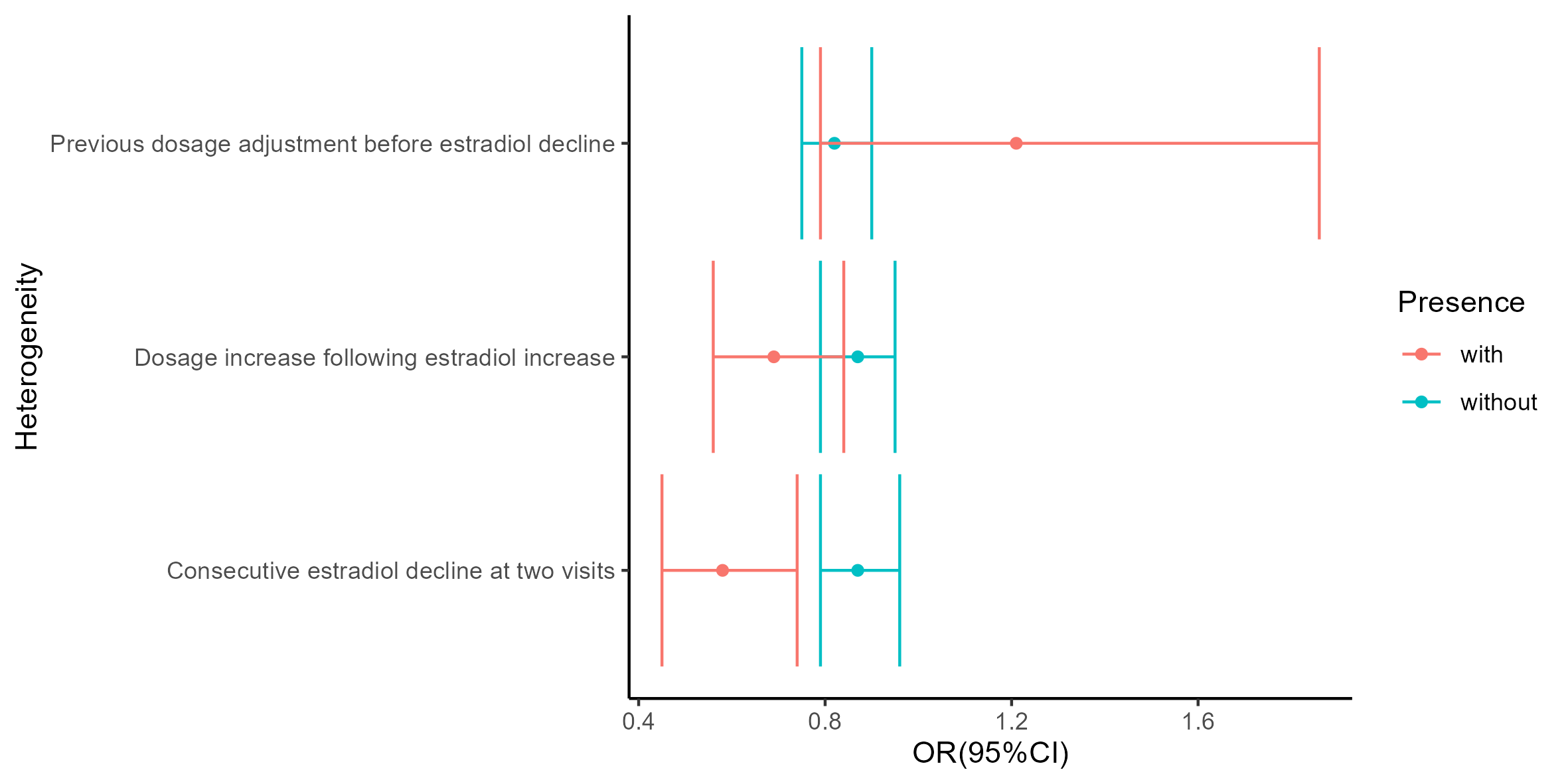

Supplement: Supplementary Figure 6 — Odds ratios (OR) comparing E2 decline subgroups with patients without E2 decline. All models are adjusted for female age and BMI, fertility-related diagnosis (duration of infertility, tubal factor, endometriosis, PCOS), ovarian reserve markers (basal FSH, LH, and AFC), and ovarian stimulation (protocol and starting dosage), male age, BMI, total motile sperm count, sperm normal morphology, insemination protocols (ICSI versus IVF), and clinical decisions (freeze-all and blastocyst culture). [file Image6.tiff]

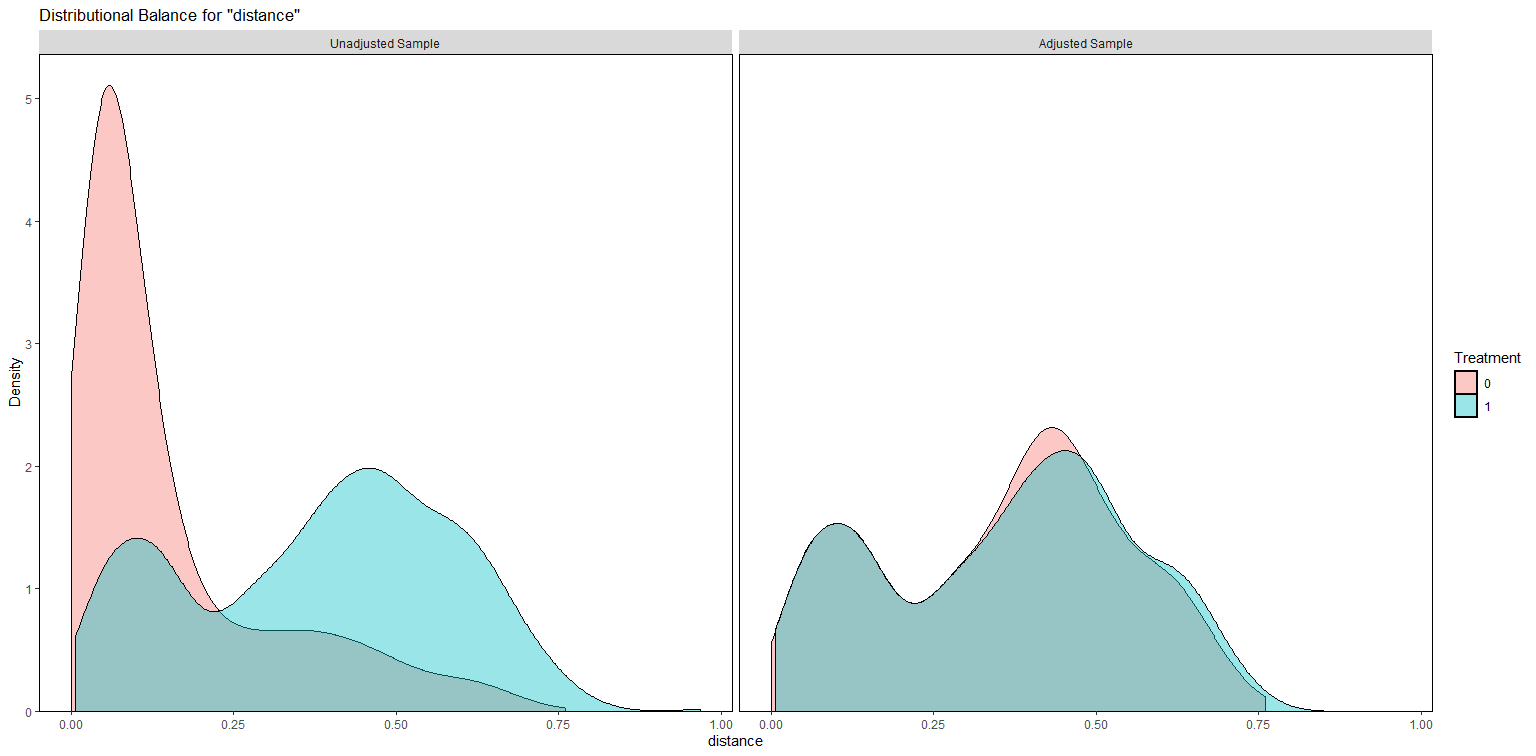

Supplement: Supplementary Figure 7 — The distribution of propensity score in patients matched for gonadotropin (Gn) increase following E2 decline. The green shades indicate patients with Gn increase and the pink shades indicate control patients with E2 decline. [file Image7.tiff]

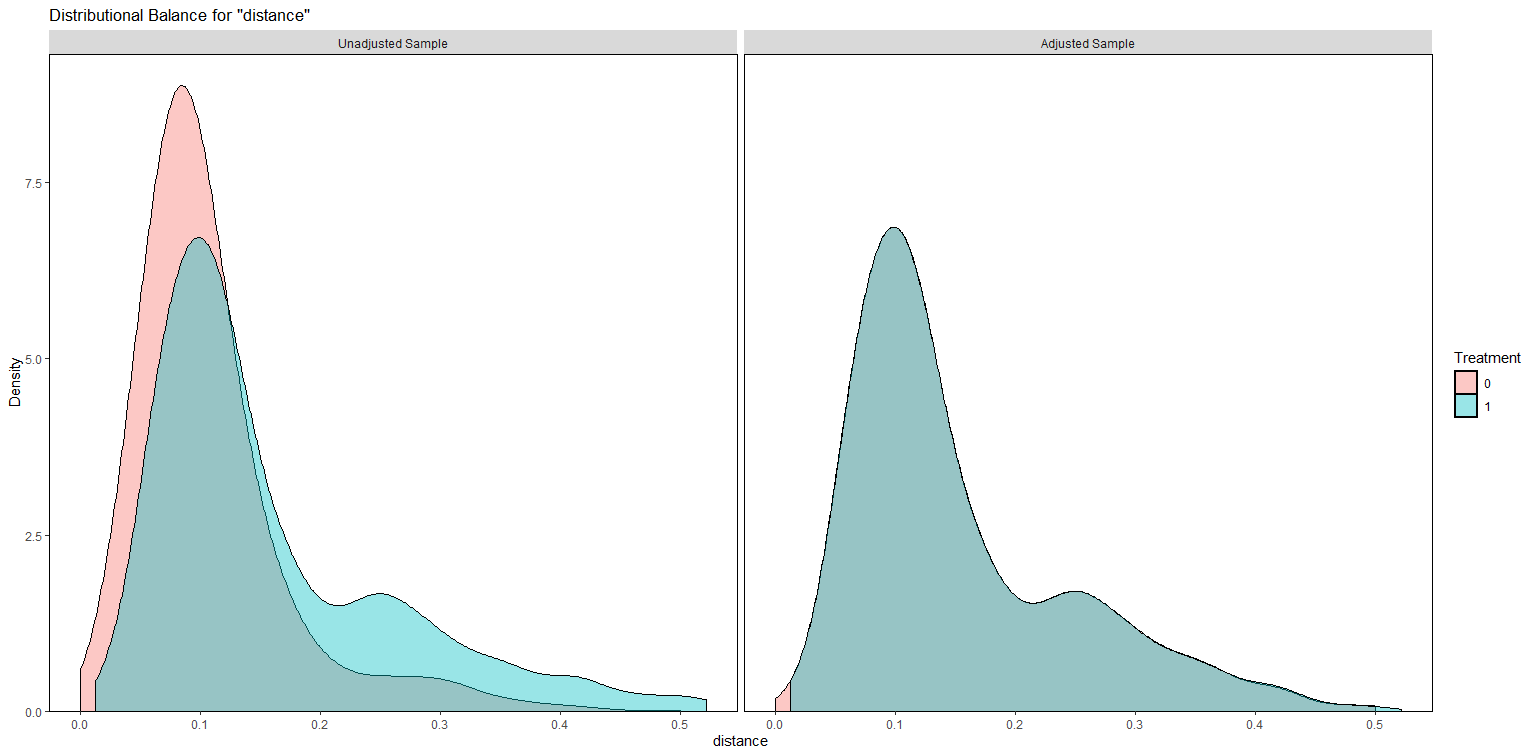

Supplement: Supplementary Figure 8 — The distribution of propensity score in patients matched for consecutive E2 decline at two visits. The green shades indicate patients with consecutive E2 decline and the pink shades indicate control patients with E2 decline. [file Image8.tiff]

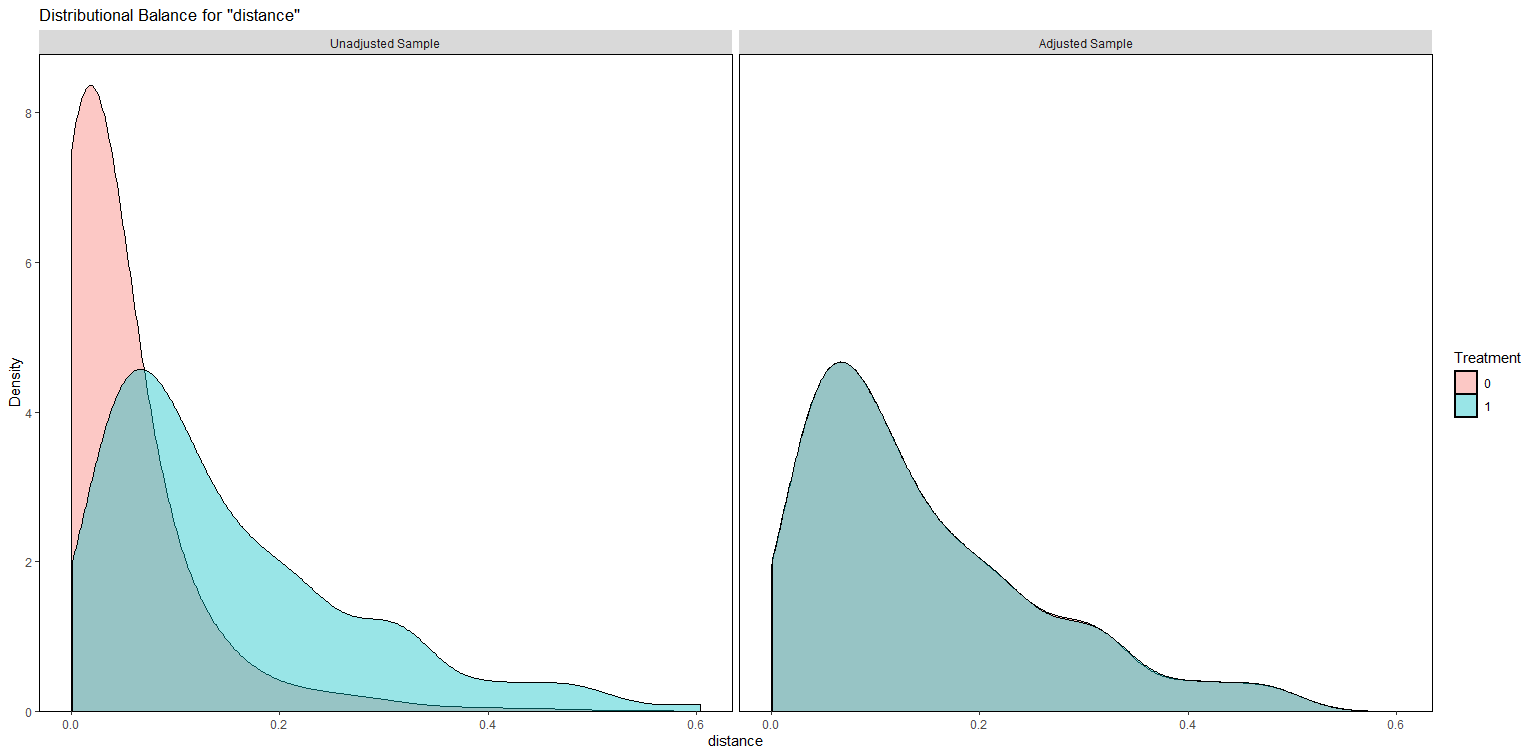

Supplement: Supplementary Figure 9 — The distribution of propensity score in patients matched for gonadotropin (Gn) adjustment before E2 decline. The green shades indicate patients with Gn adjustment and the pink shades indicate control patients with E2 decline. [file Image9.tiff]

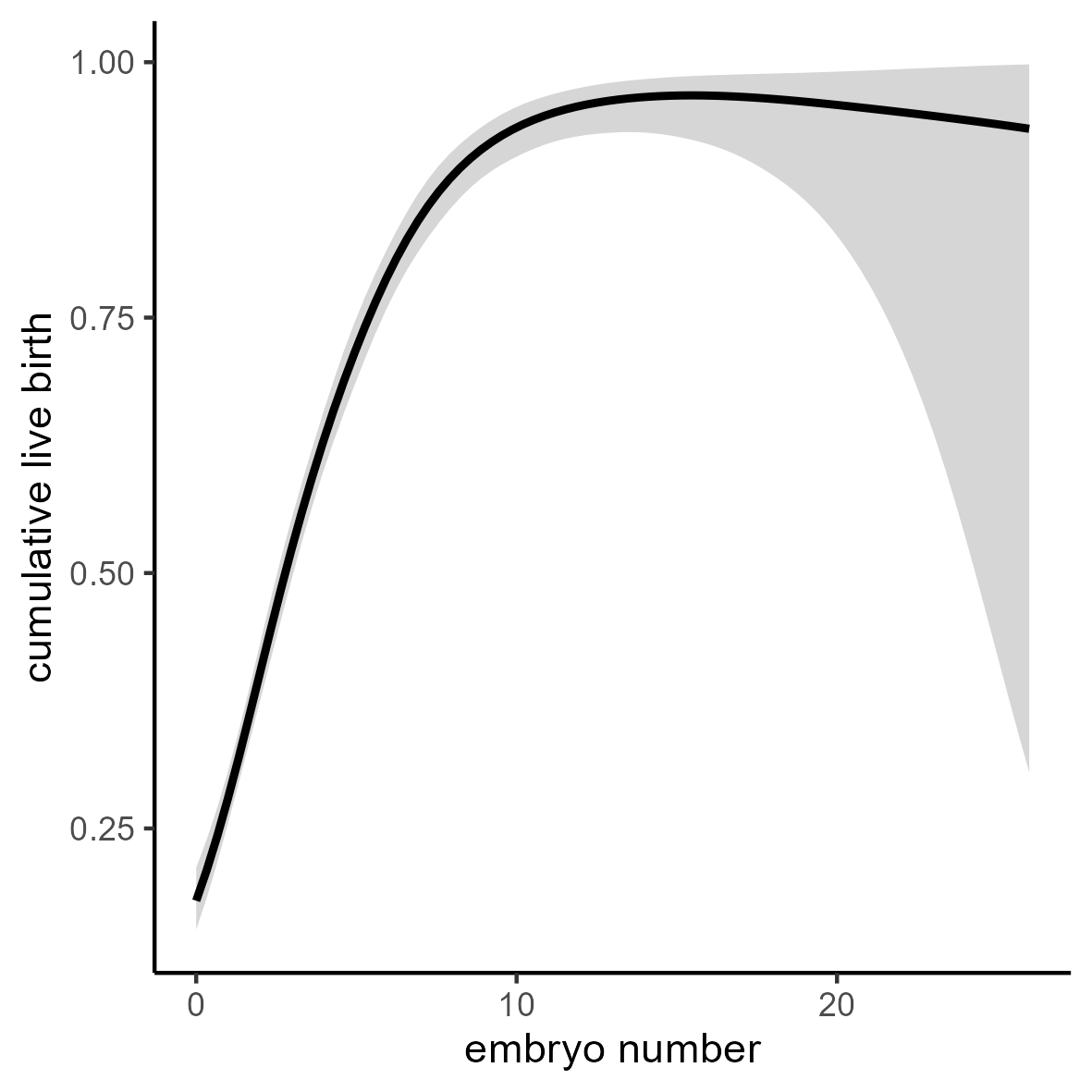

Supplement: Supplementary Figure 10 — The association between embryo yield and cumulative birth. [file Image10.tiff]
