# Supplementary material for: Unexpected estradiol decline during ovarian stimulation monitoring affects cumulative live birth
Source: Front Endocrinol (Lausanne). 2025 Oct 20;16:1658236. doi: 10.3389/fendo.2025.1658236 (PMC12580126; doi:10.3389/fendo.2025.1658236)
Supplement: Supplementary file 11 [file Table1.docx]

TableS1 Analysis of clinical data between the patients with and without Gn increase following E_2_ decrease

|  | **Unmatched** | | ***D** | **Matched** | | ***D** |
| --- | --- | --- | --- | --- | --- | --- |
|  | **Non increased** | **Increased** |  | **Non increased** | **Increased** |  |
|  | **(N=2325)** | **(N=538)** |  | **(N=493)** | **(N=493)** |  |
| **Female age, years** |  |  |  |  |  |  |
| Mean(SD) | 33.0(4.86) | 30.8(4.38) | -0.5006 | 30.7(3.99) | 30.8(4.41) | 0.0329 |
| Median[Q1,Q3] | 33.0[29.0,36.0] | 30.0[28.0,33.0] |  | 30.0[28.0,33.0] | 30.0[28.0,33.0] |  |
| **BMI, kg/m^2^** |  |  |  |  |  |  |
| Mean(SD) | 21.4(2.22) | 21.8(2.12) | 0.153 | 21.7(2.21) | 21.7(2.11) | 0.0124 |
| Median[Q1,Q3] | 21.5[19.9,23.1] | 22.1[20.2,23.4] |  | 21.7[20.2,23.2] | 21.9[20.2,23.3] |  |
| **History of live birth** | 535 (23.0%) | 89 (16.5%) | -0.0647 | 93 (18.9%) | 85 (17.2%) | -0.0162 |
| **Duration of infertility, years** |  |  |  |  |  |  |
| Mean(SD) | 4.44(3.37) | 4.10(2.91) | -0.115 | 4.08(2.88) | 4.08(2.86) | -0.0013 |
| Median[Q1,Q3] | 3.70[2.00,6.00] | 3.60[2.00,5.18] |  | 3.50[2.00,5.20] | 3.60[2.00,5.00] |  |
| **Tubal factor** | 1542 (66.3%) | 337 (62.6%) | -0.0368 | 294 (59.6%) | 307 (62.3%) | 0.0264 |
| **Polycystic ovarian syndrome** | 177 (7.6%) | 144 (26.8%) | 0.1915 | 129 (26.2%) | 127 (25.8%) | -0.0041 |
| **Endometriosis** | 233 (10.0%) | 34 (6.3%) | -0.037 | 29 (5.9%) | 33 (6.7%) | 0.0081 |
| **Basal FSH ,mIU/mL** |  |  |  |  |  |  |
| Mean(SD) | 8.82(25.1) | 17.4(231) | 0.0371 | 9.40(54.0) | 7.47(2.45) | -0.0083 |
| Median[Q1,Q3] | 7.48[6.24,9.56] | 6.96[5.86,8.32] |  | 6.53[5.56,7.79] | 7.01[5.90,8.32] |  |
| **Basal LH ,mIU/mL** |  |  |  |  |  |  |
| Mean(SD) | 4.88(3.28) | 6.33(4.21) | 0.3444 | 6.33(4.34) | 6.20(4.01) | -0.0305 |
| Median[Q1,Q3] | 4.20[3.08,5.63] | 5.12[3.76,7.71] |  | 5.01[3.60,7.70] | 5.07[3.75,7.52] |  |
| **AFC** |  |  |  |  |  |  |
| Mean(SD) | 9.20(5.89) | 15.2(6.99) | 0.8514 | 14.9(6.50) | 14.8(6.99) | -0.0131 |
| Median[Q1,Q3] | 8.00[5.00,12.0] | 15.0[10.0,20.0] |  | 15.0[10.0,20.0] | 15.0[10.0,20.0] |  |
| **Agonist protocol** | 1189 (51.1%) | 477 (88.7%) | 0.3752 | 428 (86.8%) | 432 (87.6%) | 0.0081 |
| **Antagonist protocol** | 1136 (48.9%) | 61 (11.3%) |  | 65 (13.2%) | 61 (12.4%) |  |
| **Gn starting dose,IU** |  |  |  |  |  |  |
| Mean(SD) | 206(38.1) | 156(42.2) | -1.1825 | 160(40.9) | 160(41.8) | -0.0006 |
| Median[Q1,Q3] | 225[188,225] | 150[113,188] |  | 150[113,188] | 150[113,188] |  |
| **IVF** | 1694 (72.9%) | 415 (77.1%) |  | 359 (72.8%) | 375 (76.1%) |  |
| **ICSI** | 631 (27.1%) | 123 (22.9%) | -0.0428 | 134 (27.2%) | 118 (23.9%) | -0.0325 |
| **Whole embryo blastocyst culture** | 460 (19.8%) | 147 (27.3%) | 0.0754 | 134 (27.2%) | 131 (26.6%) | -0.0061 |
| **Freeze-all** | 420 (18.1%) | 90 (16.7%) | -0.0134 | 93 (18.9%) | 87 (17.6%) | -0.0122 |
| **Male age,years** |  |  |  |  |  |  |
| Mean(SD) | 34.5(5.46) | 32.5(4.91) | -0.4174 | 32.3(4.38) | 32.6(4.96) | 0.0476 |
| Median[Q1,Q3] | 34.0[30.0,38.0] | 32.0[29.0,35.0] |  | 32.0[29.0,35.0] | 32.0[29.0,35.0] |  |
| **Male BMI ,kg/m^2^** |  |  |  |  |  |  |
| Mean(SD) | 24.0(3.36) | 24.0(3.81) | -0.004 | 23.8(3.66) | 23.9(3.76) | 0.0266 |
| Median[Q1,Q3] | 23.9[21.7,26.1] | 23.7[21.3,26.0] |  | 23.6[21.3,26.0] | 23.7[21.3,25.9] |  |
| **Sperm normal morphology,%** |  |  |  |  |  |  |
| Mean(SD) | 7.15(4.60) | 7.50(6.11) | 0.0573 | 7.24(4.90) | 7.49(6.26) | 0.0415 |
| Median[Q1,Q3] | 6.00[4.00,9.00] | 6.00[4.00,9.48] |  | 6.00[4.00,9.00] | 6.00[4.00,9.00] |  |
| **Total motile sperm count,10^6^/ml** |  |  |  |  |  |  |
| Mean(SD) | 74.5(527) | 68.4(69.5) | -0.0869 | 66.9(68.4) | 68.3(70.1) | 0.0204 |
| Median[Q1,Q3] | 44.2[17.0,86.2] | 47.9[19.3,96.0] |  | 45.8[17.5,92.9] | 47.1[19.5,96.9] |  |

Data were presented as mean±SD and median [first quartile, third quartile] for continuous variables and n (percentage) for categorical variables. *D: Standardized difference. The absolute value of D is less than 0.1, cohorts can be considered to be balanced concerning the demographics being assessed.

TableS2 The Impact of Gn increase following E_2_ decrease on Pregnancy Outcomes

|  | **Unmatched** | | **P-value** | **Matched** | | **P-value** |
| --- | --- | --- | --- | --- | --- | --- |
|  | **Non increased** | **Increased** |  | **Non increased** | **Increased** |  |
|  | **(N=2325)** | **(N=538)** |  | **(N=493)** | **(N=493)** |  |
| **Absolute E2 decline, pg/ml** |  |  |  |  |  |  |
| Mean(SD) | -262(476) | -167(395) | <0.001 | -387(688) | -171(399) | <0.001 |
| Median[Q1,Q3] | -95.3[-289,-27.0] | -24.5[-136,-8.00] |  | -139[-459,-38.0] | -26.0[-145,-8.00] |  |
| **Percentage of E2 decline, %** |  |  |  |  |  |  |
| Mean(SD) | 18.2(20.5) | 30.3(24.0) | <0.001 | 21.4(21.3) | 29.2(22.9) | <0.001 |
| Median[Q1,Q3] | 10.9[4.49,23.6] | 25.0[10.3,44.6] |  | 14.4[5.36,29.5] | 24.6[10.2,43.2] |  |
| **Consecutive decline in the next visit** | 236 (10.2%) | 89 (16.5%) | <0.001 | 60 (12.2%) | 85 (17.2%) | 0.031 |
| **Dosage change following the decline, IU** |  |  |  |  |  |  |
| Mean(SD) | -0.505(5.35) | 54.7(21.8) | <0.001 | -0.532(5.04) | 54.8(21.8) | <0.001 |
| Median[Q1,Q3] | 0[0,0] | 37.5[37.5,75.0] |  | 0[0,0] | 37.5[37.5,75.0] |  |
| **Follicular diameter changes at the decline** |  |  |  |  |  |  |
| Mean(SD) | 1.92(2.26) | 1.34(1.99) | <0.001 | 1.91(2.10) | 1.33(2.02) | <0.001 |
| Median[Q1,Q3] | 1.60[0.800,2.80] | 1.30[0.500,2.48] |  | 1.50[0.500,2.90] | 1.30[0.500,2.40] |  |
| **Dosage used when the decline occurs, IU** |  |  |  |  |  |  |
| Mean(SD) | 122(109) | 212(53.8) | <0.001 | 89.8(94.2) | 215(54.4) | <0.001 |
| Median[Q1,Q3] | 150[0,225] | 225[188,225] |  | 100[0,188] | 225[188,225] |  |
| **Follicle count changes at the decline** |  |  |  |  |  |  |
| Mean(SD) | 0.162(2.12) | -1.56(4.05) | <0.001 | -0.128(3.21) | -1.37(3.65) | <0.001 |
| Median[Q1,Q3] | 0[-1.00,1.00] | -1.00[-4.00,0] |  | 0[-1.00,1.00] | -1.00[-3.00,1.00] |  |
| **Dosage adjustment before the decline, IU** |  |  |  |  |  |  |
| Mean(SD) | -0.253(15.7) | 0.651(21.8) | 0.00157 | 0.380(21.2) | 0.102(22.0) | 0.247 |
| Median[Q1,Q3] | 0[0,0] | 0[0,0] |  | 0[0,0] | 0[0,0] |  |
| **Dosage adjustment before the decline, IU** | 104 (4.5%) | 42 (7.8%) | 0.00222 | 46 (9.3%) | 41 (8.3%) | 0.653 |
| **Total usage of Gn, IU** |  |  |  |  |  |  |
| Mean(SD) | 2480(847) | 3140(1120) | <0.001 | 2520(954) | 3160(1140) | <0.001 |
| Median[Q1,Q3] | 2250[1800,2930] | 2930[2330,3730] |  | 2290[1800,3080] | 2950[2330,3750] |  |
| **Gn medication duration** |  |  |  |  |  |  |
| Mean(SD) | 11.9(3.78) | 16.7(4.37) | <0.001 | 14.4(4.19) | 16.6(4.39) | <0.001 |
| Median[Q1,Q3] | 11.0[9.00,14.0] | 16.0[14.0,19.0] |  | 14.0[11.0,17.0] | 16.0[14.0,19.0] |  |
| **Estradiol on HCG day, pg/ml** |  |  |  |  |  |  |
| Mean(SD) | 2130(1820) | 2590(2050) | <0.001 | 2620(1990) | 2600(2090) | 0.571 |
| Median[Q1,Q3] | 1520[839,3030] | 2030[1110,3540] |  | 2050[1130,3890] | 2030[1120,3510] |  |
| **LH on HCG day ,mIU/mL** |  |  |  |  |  |  |
| Mean(SD) | 1.68(2.57) | 1.05(1.41) | <0.001 | 0.927(1.17) | 1.07(1.44) | 0.197 |
| Median[Q1,Q3] | 1.00[0.510,1.99] | 0.605[0.360,1.09] |  | 0.590[0.340,1.04] | 0.620[0.370,1.13] |  |
| **P on HCG day,ng/ml** |  |  |  |  |  |  |
| Mean(SD) | 0.960(1.29) | 0.867(0.520) | 0.229 | 0.838(0.588) | 0.875(0.528) | 0.03 |
| Median[Q1,Q3] | 0.780[0.510,1.16] | 0.755[0.523,1.06] |  | 0.680[0.460,1.07] | 0.760[0.530,1.10] |  |
| **Oocyte yield** |  |  |  |  |  |  |
| Mean(SD) | 6.96(5.44) | 7.92(5.47) | <0.001 | 8.87(6.11) | 7.82(5.49) | 0.007 |
| Median[Q1,Q3] | 5.00[3.00,10.0] | 7.00[4.00,11.0] |  | 8.00[4.00,12.0] | 6.00[4.00,11.0] |  |
| **Mature oocyte** |  |  |  |  |  |  |
| Mean(SD) | 6.20(5.01) | 7.02(5.08) | <0.001 | 7.85(5.70) | 6.93(5.10) | 0.0128 |
| Median[Q1,Q3] | 5.00[3.00,8.00] | 6.00[3.00,10.0] |  | 7.00[4.00,11.0] | 6.00[3.00,10.0] |  |
| **Oocyte maturation rate, %** |  |  |  |  |  |  |
| Mean(SD) | 88.5(20.0) | 88.1(17.6) | 0.0214 | 88.0(17.9) | 88.3(17.4) | 0.793 |
| Median[Q1,Q3] | 100[83.3,100] | 100[80.0,100] |  | 100[80.0,100] | 100[80.0,100] |  |
| **Zygote** |  |  |  |  |  |  |
| Mean(SD) | 5.49(4.53) | 6.18(4.61) | <0.001 | 6.89(5.08) | 6.10(4.62) | 0.014 |
| Median[Q1,Q3] | 4.00[2.00,8.00] | 5.00[3.00,8.00] |  | 6.00[3.00,9.00] | 5.00[3.00,8.00] |  |
| **Fertility rate, %** |  |  |  |  |  |  |
| Mean(SD) | 79.5(25.0) | 78.5(23.2) | 0.046 | 78.5(23.2) | 78.6(23.0) | 0.865 |
| Median[Q1,Q3] | 87.5[66.7,100] | 83.3[66.7,100] |  | 83.3[66.7,100] | 83.3[66.7,100] |  |
| **2 Pronuclei embryo** |  |  |  |  |  |  |
| Mean(SD) | 4.39(3.79) | 4.92(3.85) | <0.001 | 5.55(4.36) | 4.85(3.85) | 0.015 |
| Median[Q1,Q3] | 3.00[2.00,6.00] | 4.00[2.00,6.00] |  | 5.00[2.00,7.00] | 4.00[2.00,6.00] |  |
| **Cleavage** |  |  |  |  |  |  |
| Mean(SD) | 4.67(3.97) | 5.32(4.07) | <0.001 | 5.91(4.49) | 5.26(4.08) | 0.025 |
| Median[Q1,Q3] | 4.00[2.00,6.00] | 4.00[2.00,7.00] |  | 5.00[2.00,8.00] | 4.00[2.00,7.00] |  |
| **Cleavage 2 pronuclei embryo** |  |  |  |  |  |  |
| Mean(SD) | 4.30(3.74) | 4.83(3.76) | <0.001 | 5.46(4.32) | 4.77(3.77) | 0.018 |
| Median[Q1,Q3] | 3.00[2.00,6.00] | 4.00[2.00,6.00] |  | 4.00[2.00,7.00] | 4.00[2.00,6.00] |  |
| **No embryo** | 126 (5.4%) | 20 (3.7%) | 0.131 | 15 (3.0%) | 19 (3.9%) | 0.601 |
| **Good quality embryos** |  |  |  |  |  |  |
| Mean(SD) | 2.63(2.82) | 3.13(2.89) | <0.001 | 3.37(3.14) | 3.07(2.89) | 0.217 |
| Median[Q1,Q3] | 2.00[1.00,4.00] | 2.00[1.00,4.00] |  | 3.00[1.00,5.00] | 2.00[1.00,4.00] |  |
| **Good quality embryo rate, %** |  |  |  |  |  |  |
| Mean(SD) | 49.8(36.3) | 54.2(32.2) | 0.0265 | 52.6(32.7) | 53.5(32.4) | 0.719 |
| Median[Q1,Q3] | 50.0[11.1,80.0] | 57.1[33.3,77.8] |  | 57.1[28.6,78.6] | 55.0[33.3,75.0] |  |
| **Cumulative live birth rate, %** | 1213 (52.2%) | 363 (67.5%) | <0.001 | 331 (67.1%) | 331 (67.1%) | ＞0.999 |
| **Fresh embryo transfer,N** | **(N=1779)** | **(N=428)** |  | **(N=385)** | **(N=387)** |  |
| **Fresh embryo transfer number** |  |  |  |  |  |  |
| 1 | 715 (40.2%) | 197 (46.0%) | 0.006 | 156 (40.5%) | 169 (43.7%) | 0.505 |
| 2 | 1020 (57.3%) | 229 (53.5%) |  | 225 (58.4%) | 216 (55.8%) |  |
| 3 | 44 (2.5%) | 2 (0.5%) |  | 4 (1.0%) | 2 (0.5%) |  |
| **Fresh blastocyst transfer** | 162 (9.1%) | 71 (16.6%) | <0.001 | 58 (15.1%) | 57 (14.7%) | 0.976 |
| **Fresh cycle live birth** | 784 (44.1%) | 257 (60.0%) | <0.001 | 222 (57.7%) | 231 (59.7%) | 0.618 |
| **aOR (95% CI)*** | ref | 1.04(0.8,1.35) | 0.78 | ref | 1.12(0.82,1.52) | 0.484 |

All models were adjusted for female and male age, BMI, history of live birth, duration of infertility, tubal factor, PCOS, endometriosis, basic FSH, LH, antral follicle, agonist protocol, Antagonist protocol, Gn start dose, IVF, ICSI, whole embryo blastocyst culture, freeze-all, Sperm normal morphology, total mobil sperm count. The cumulative live birth rate was the dependent variable.

TableS3 Analysis of clinical data between the single E_2_ decrease group and the multiple continuous E_2_ decrease group.

|  | **Unmatched** | | ***D** | **Matched** | | ***D** |
| --- | --- | --- | --- | --- | --- | --- |
|  | **Single E_2_ decrease group** | **Multiple continuous E2 decrease group** |  | **Single E_2_ decrease group** | **Multiple continuous E_2_ decrease group** |  |
|  | **(N=2538)** | **(N=325)** |  | **(N=322)** | **(N=322)** |  |
| **Female age ,years** |  |  |  |  |  |  |
| Mean(SD) | 32.6(4.82) | 32.4(5.10) | -0.0268 | 32.6(4.93) | 32.5(5.10) | -0.0268 |
| Median[Q1,Q3] | 32.0[29.0,36.0] | 32.0[28.0,36.0] |  | 32.0[29.0,36.0] | 32.0[28.0,36.0] |  |
| **BMI,kg/m^2^** |  |  |  |  |  |  |
| Mean(SD) | 21.4(2.19) | 21.9(2.22) | 0.0159 | 21.9(2.03) | 21.9(2.22) | 0.0159 |
| Median[Q1,Q3] | 21.6[19.9,23.1] | 22.0[20.3,23.8] |  | 22.1[20.5,23.4] | 22.0[20.3,23.8] |  |
| **History of live birth** | 558 (22.0%) | 66 (20.3%) | -0.0373 | 78 (24.2%) | 66 (20.5%) | -0.0373 |
| **Duration of infertility ,years** |  |  |  |  |  |  |
| Mean(SD) | 4.36(3.32) | 4.52(3.07) | 0.0736 | 4.28(3.46) | 4.51(3.08) | 0.0736 |
| Median[Q1,Q3] | 3.50[2.00,6.00] | 4.00[2.00,6.00] |  | 3.30[2.00,5.80] | 4.00[2.00,6.00] |  |
| **Tubal factor** | 1674 (66.0%) | 205 (63.1%) | -0.0186 | 208 (64.6%) | 202 (62.7%) | -0.0186 |
| **Polycystic ovarian syndrome** | 235 (9.3%) | 86 (26.5%) | 0.0062 | 81 (25.2%) | 83 (25.8%) | 0.0062 |
| **Endometriosis** | 239 (9.4%) | 28 (8.6%) | 0 | 28 (8.7%) | 28 (8.7%) | 0 |
| **Basal FSH ,mIU/mL** |  |  |  |  |  |  |
| Mean(SD) | 10.7(109) | 8.46(3.95) | 0.1207 | 8.02(3.26) | 8.50(3.95) | 0.1207 |
| Median[Q1,Q3] | 7.38[6.15,9.25] | 7.43[6.17,9.98] |  | 7.19[5.98,9.26] | 7.44[6.17,9.99] |  |
| **Basal LH ,mIU/mL** |  |  |  |  |  |  |
| Mean(SD) | 5.05(3.44) | 5.96(4.01) | -0.0485 | 6.11(4.63) | 5.92(3.99) | -0.0485 |
| Median[Q1,Q3] | 4.30[3.14,5.82] | 4.66[3.39,7.43] |  | 4.78[3.33,7.16] | 4.65[3.39,7.41] |  |
| **AFC** |  |  |  |  |  |  |
| Mean(SD) | 10.1(6.27) | 12.2(8.13) | 0.0122 | 11.9(7.67) | 12.0(8.06) | 0.0122 |
| Median[Q1,Q3] | 9.00[5.00,14.0] | 10.0[5.00,20.0] |  | 11.0[5.00,19.0] | 10.0[5.00,20.0] |  |
| **Agonist protocol** | 1471 (58.0%) | 195 (60.0%) | -0.0031 | 193 (59.9%) | 192 (59.6%) | -0.0031 |
| **Antagonist protocol** | 1067 (42.0%) | 130 (40.0%) |  | 129 (40.1%) | 130 (40.4%) |  |
| **Gn starting dose,IU** |  |  |  |  |  |  |
| Mean(SD) | 198(42.5) | 187(49.4) | -0.0189 | 188(46.5) | 187(49.2) | -0.0189 |
| Median[Q1,Q3] | 225[150,225] | 225[150,225] |  | 225[150,225] | 225[150,225] |  |
| **IVF** | 1885 (74.3%) | 224 (68.9%) |  | 220 (68.3%) | 222 (68.9%) |  |
| **ICSI** | 653 (25.7%) | 101 (31.1%) | -0.0062 | 102 (31.7%) | 100 (31.1%) | -0.0062 |
| **Whole embryo blastocyst culture** | 541 (21.3%) | 66 (20.3%) | -0.028 | 73 (22.7%) | 64 (19.9%) | -0.028 |
| **Freeze-all** | 448 (17.7%) | 62 (19.1%) | -0.0155 | 65 (20.2%) | 60 (18.6%) | -0.0155 |
| **Male age,years** |  |  |  |  |  |  |
| Mean(SD) | 34.1(5.37) | 34.3(5.80) | 0.0182 | 34.2(5.56) | 34.3(5.81) | 0.0182 |
| Median[Q1,Q3] | 33.0[30.0,38.0] | 33.0[30.0,38.0] |  | 33.0[30.0,38.0] | 33.5[30.0,38.0] |  |
| **Male BMI ,kg/m^2^** |  |  |  |  |  |  |
| Mean(SD) | 24.0(3.42) | 23.9(3.67) | -0.0556 | 24.1(3.49) | 23.9(3.67) | -0.0556 |
| Median[Q1,Q3] | 23.9[21.6,26.0] | 23.8[21.5,26.1] |  | 24.0[22.0,26.0] | 23.8[21.5,26.1] |  |
| **Sperm normal morphology,%** |  |  |  |  |  |  |
| Mean(SD) | 7.23(4.99) | 7.13(4.41) | 0.0078 | 7.10(4.57) | 7.14(4.42) | 0.0078 |
| Median[Q1,Q3] | 6.00[4.00,9.00] | 6.00[4.00,9.00] |  | 6.50[4.00,8.50] | 6.00[4.00,9.00] |  |
| **Sperm motility,10^6^/ml** |  |  |  |  |  |  |
| Mean(SD) | 43.3(20.0) | 44.4(19.7) | #N/A | 41.7(19.6) | 44.5(19.7) | #N/A |
| Median[Q1,Q3] | 45.3[29.9,56.7] | 47.4[31.2,57.0] |  | 43.0[27.8,54.5] | 47.4[31.2,57.0] |  |
| **Total motile sperm count,10^6^/ml** |  |  |  |  |  |  |
| Mean(SD) | 74.7(505) | 62.8(64.9) | 0.0482 | 60.1(61.4) | 63.2(65.0) | 0.0482 |
| Median[Q1,Q3] | 44.8[17.2,88.4] | 44.0[15.2,85.9] |  | 40.6[15.6,81.7] | 45.4[15.2,86.0] |  |

Data were presented as mean±SD and median [first quartile, third quartile] for continuous variables and n (percentage) for categorical variables. *D: Standardized difference. The absolute value of D is less than 0.1, cohorts can be considered to be balanced concerning the demographics being assessed.

TableS4 The Impact of single E_2_ decrease and multiple continuous E_2_ decrease on pregnancy outcomes

|  | **Unmatched** | | **P-value** | **matched** | | **P-value** |
| --- | --- | --- | --- | --- | --- | --- |
|  | **Single E_2_ decrease group** | **Multiple continuous E2 decrease group** |  | **Single E_2_ decrease group** | **Multiple continuous E_2_ decrease group** |  |
|  | **(N=2538)** | **(N=325)** |  | **(N=322)** | **(N=322)** |  |
| **Absolute E2 decline, pg/ml** |  |  |  |  |  |  |
| Mean(SD) | -228(446) | -368(562) | <0.001 | -182(328) | -370(564) | <0.001 |
| Median[Q1,Q3] | -75.0[-241,-17.0] | -151[-468,-54.0] |  | -54.5[-186,-13.0] | -152[-470,-54.3] |  |
| **Percentage of E2 decline, %** |  |  |  |  |  |  |
| Mean(SD) | 18.7(20.5) | 34.2(25.9) | <0.001 | 21.0(20.7) | 34.4(24.9) | <0.001 |
| Median[Q1,Q3] | 11.4[4.66,25.0] | 27.6[13.0,49.8] |  | 14.4[6.33,28.4] | 27.8[14.6,50.2] |  |
| **Dosage change following the decline, IU** |  |  |  |  |  |  |
| Mean(SD) | 9.37(23.1) | 13.8(30.2) | <0.001 | 14.4(26.3) | 13.7(30.2) | 0.69 |
| Median[Q1,Q3] | 0[0,0] | 0[0,37.5] |  | 0[0,37.5] | 0[0,37.5] |  |
| **Follicular diameter changes at the decline** |  |  |  |  |  |  |
| Mean(SD) | 1.86(2.20) | 1.43(2.36) | <0.001 | 1.64(2.24) | 1.44(2.36) | 0.007 |
| Median[Q1,Q3] | 1.50[0.700,2.70] | 1.10[0.200,2.50] |  | 1.50[0.600,2.50] | 1.10[0.250,2.50] |  |
| **Dosage used when the decline occurs, IU** |  |  |  |  |  |  |
| Mean(SD) | 135(108) | 168(94.9) | <0.001 | 151(99.5) | 168(95.3) | 0.056 |
| Median[Q1,Q3] | 188[0,225] | 225[113,225] |  | 188[0,225] | 225[113,225] |  |
| **Follicle count changes at the decline** |  |  |  |  |  |  |
| Mean(SD) | -0.120(2.57) | -0.477(3.39) | 0.002 | -0.345(3.83) | -0.460(3.40) | 0.132 |
| Median[Q1,Q3] | 0[-1.00,1.00] | 0[-1.00,1.00] |  | 0[-1.00,1.00] | 0[-1.00,1.00] |  |
| **Dosage adjustment before the decline, IU** |  |  |  |  |  |  |
| Mean(SD) | -0.251(16.7) | 1.23(19.4) | 0.163 | -2.25(17.8) | 1.48(19.2) | 0.02 |
| Median[Q1,Q3] | 0[0,0] | 0[0,0] |  | 0[0,0] | 0[0,0] |  |
| **Dosage decrease before the decline (%)** | 122 (4.8%) | 24 (7.4%) | 0.064 | 26 (8.1%) | 22 (6.8%) | 0.653 |
| **Total dosage of Gn, IU** |  |  |  |  |  |  |
| Mean(SD) | 2530(876) | 3140(1220) | <0.001 | 2560(957) | 3140(1220) | <0.001 |
| Median[Q1,Q3] | 2330[1910,2930] | 2890[2250,3940] |  | 2340[1810,2930] | 2850[2250,3940] |  |
| **Gn medication duration** |  |  |  |  |  |  |
| Mean(SD) | 12.4(4.00) | 15.8(5.51) | <0.001 | 13.0(4.53) | 15.7(5.49) | <0.001 |
| Median[Q1,Q3] | 12.0[9.00,15.0] | 15.0[11.0,20.0] |  | 12.0[9.00,16.0] | 15.0[11.0,20.0] |  |
| **Estradiol on HCG day ,pg/mL** |  |  |  |  |  |  |
| Mean(SD) | 2260(1880) | 1860(1800) | <0.001 | 2230(2000) | 1850(1790) | <0.001 |
| Median[Q1,Q3] | 1650[923,3260] | 1190[690,2490] |  | 1640[869,2790] | 1190[686,2480] |  |
| **LH on HCG day ,mIU/mL** |  |  |  |  |  |  |
| Mean(SD) | 1.54(1.85) | 1.69(4.93) | 0.006 | 1.58(1.76) | 1.69(4.95) | 0.035 |
| Median[Q1,Q3] | 0.900[0.480,1.89] | 0.760[0.370,1.66] |  | 0.970[0.463,1.91] | 0.765[0.373,1.65] |  |
| **P on HCG day,ng/mL** |  |  |  |  |  |  |
| Mean(SD) | 0.930(0.645) | 1.04(3.02) | 0.009 | 0.880(0.682) | 1.04(3.03) | 0.743 |
| Median[Q1,Q3] | 0.790[0.530,1.15] | 0.690[0.450,1.16] |  | 0.720[0.490,1.03] | 0.685[0.450,1.16] |  |
| **Oocyte yield** |  |  |  |  |  |  |
| Mean(SD) | 7.26(5.45) | 6.24(5.45) | <0.001 | 7.56(5.97) | 6.16(5.38) | <0.001 |
| Median[Q1,Q3] | 6.00[3.00,10.0] | 5.00[2.00,8.00] |  | 6.00[3.00,10.0] | 5.00[2.00,8.00] |  |
| **Mature oocyte** |  |  |  |  |  |  |
| Mean(SD) | 6.46(5.02) | 5.50(5.06) | <0.001 | 6.74(5.50) | 5.46(5.05) | <0.001 |
| Median[Q1,Q3] | 5.00[3.00,9.00] | 4.00[2.00,7.00] |  | 5.00[3.00,9.00] | 4.00[2.00,7.00] |  |
| **Oocyte maturation rate, %** |  |  |  |  |  |  |
| Mean(SD) | 88.7(18.9) | 86.2(23.8) | 0.816 | 88.1(19.7) | 86.3(23.9) | 0.468 |
| Median[Q1,Q3] | 100[83.3,100] | 100[80.0,100] |  | 100[80.3,100] | 100[80.0,100] |  |
| **Zygote** |  |  |  |  |  |  |
| Mean(SD) | 5.71(4.54) | 4.94(4.64) | <0.001 | 5.76(4.83) | 4.91(4.64) | 0.006 |
| Median[Q1,Q3] | 5.00[2.00,8.00] | 4.00[2.00,7.00] |  | 4.00[2.00,8.00] | 4.00[2.00,7.00] |  |
| **Fertility rate, %** |  |  |  |  |  |  |
| Mean(SD) | 79.5(24.1) | 77.8(28.7) | 0.732 | 76.5(25.9) | 78.0(28.7) | 0.066 |
| Median[Q1,Q3] | 85.7[66.7,100] | 87.5[66.7,100] |  | 82.6[66.7,100] | 88.0[66.7,100] |  |
| **2 pronuclei embryo** |  |  |  |  |  |  |
| Mean(SD) | 4.57(3.80) | 3.87(3.77) | <0.001 | 4.65(4.10) | 3.85(3.77) | 0.005 |
| Median[Q1,Q3] | 4.00[2.00,6.00] | 3.00[1.00,5.00] |  | 3.00[2.00,7.00] | 3.00[1.00,5.00] |  |
| **Cleavage** |  |  |  |  |  |  |
| Mean(SD) | 4.87(3.98) | 4.22(4.09) | <0.001 | 4.92(4.26) | 4.20(4.09) | 0.011 |
| Median[Q1,Q3] | 4.00[2.00,7.00] | 3.00[1.00,6.00] |  | 4.00[2.00,7.00] | 3.00[1.25,5.00] |  |
| **Cleavage 2 pronuclei embryo** |  |  |  |  |  |  |
| Mean(SD) | 4.48(3.75) | 3.80(3.70) | <0.001 | 4.57(4.04) | 3.79(3.69) | 0.006 |
| Median[Q1,Q3] | 4.00[2.00,6.00] | 3.00[1.00,5.00] |  | 3.00[2.00,6.00] | 3.00[1.00,5.00] |  |
| **No embryo** | 117 (4.6%) | 29 (8.9%) | 0.001 | 18 (5.6%) | 29 (9.0%) | 0.13 |
| **Good quality embryos** |  |  |  |  |  |  |
| Mean(SD) | 2.77(2.87) | 2.39(2.62) | 0.022 | 2.84(3.04) | 2.37(2.61) | 0.077 |
| Median[Q1,Q3] | 2.00[1.00,4.00] | 2.00[0,3.00] |  | 2.00[1.00,4.00] | 2.00[0,3.00] |  |
| **Good quality embryo rate, %** |  |  |  |  |  |  |
| Mean(SD) | 50.8(35.4) | 49.6(36.7) | 0.597 | 51.1(35.9) | 49.2(36.7) | 0.529 |
| Median[Q1,Q3] | 50.0[20.0,80.0] | 50.0[0,77.8] |  | 50.0[20.0,80.0] | 50.0[0,76.4] |  |
| **Cumulative live birth rate, %** | 1416 (55.8%) | 160 (49.2%) | 0.029 | 183 (56.8%) | 159 (49.4%) | 0.069 |
| **Fresh embryo transfer,N** | **(N=1973)** | **(N=234)** |  | **(N=239)** | **(N=233)** |  |
| **Fresh embryo transfer number** |  |  |  |  |  |  |
| 1 | 814 (41.3%) | 98 (41.9%) | 0.906 | 94 (39.3%) | 97 (41.6%) | 0.875 |
| 2 | 1117 (56.6%) | 132 (56.4%) |  | 141 (59.0%) | 132 (56.7%) |  |
| 3 | 42 (2.1%) | 4 (1.7%) |  | 4 (1.7%) | 4 (1.7%) |  |
| **Fresh blastocyst transfer** | 215 (10.9%) | 18 (7.7%) | 0.163 | 25 (10.5%) | 18 (7.7%) | 0.383 |
| **Fresh cycle live birth** | 939 (47.6%) | 102 (43.6%) | 0.276 | 123 (51.5%) | 102 (43.8%) | 0.114 |
| **aOR (95% CI)*** | ref | 0.8(0.59,1.09) | 0.159 | ref | 0.72(0.48,1.1) | 0.132 |

All models were adjusted for female and male age, BMI, history of live birth, duration of infertility, tubal factor, PCOS, endometriosis, basic FSH, LH, antral follicle, agonist protocol, Antagonist protocol, Gn start dose, IVF, ICSI, whole embryo blastocyst culture, freeze-all, Sperm normal morphology, total mobil sperm count. The cumulative live birth rate was the dependent variable.

TableS5 Analysis of clinical data between the patients with and without Gn decrease before E_2_ decrease

|  | **Unmatched** | | ***D** | **Matched** | | ***D** |
| --- | --- | --- | --- | --- | --- | --- |
|  | N**on decreased** | **Decreased** |  | **Non decreased** | **Decreased** |  |
|  | **(N=2717)** | **(N=146)** |  | **(N=143)** | **(N=143)** |  |
| **Female age ,years** |  |  |  |  |  |  |
| Mean(SD) | 32.7(4.85) | 29.6(3.88) | -0.7985 | 30.0(3.55) | 29.7(3.88) | -0.0703 |
| Median[Q1,Q3] | 32.0[29.0,36.0] | 29.0[27.0,32.0] |  | 30.0[27.5,32.0] | 29.0[27.0,32.0] |  |
| **BMI,kg/m^2^** |  |  |  |  |  |  |
| Mean(SD) | 21.5(2.20) | 21.1(2.24) | -0.1716 | 21.0(2.43) | 21.2(2.26) | 0.0594 |
| Median[Q1,Q3] | 21.6[20.0,23.2] | 21.1[19.5,22.8] |  | 21.1[19.2,22.9] | 21.1[19.4,22.9] |  |
| **History of live birth** | 599 (22.0%) | 25 (17.1%) | -0.0492 | 30 (21.0%) | 25 (17.5%) | -0.035 |
| **Duration of infertility ,years** |  |  |  |  |  |  |
| Mean(SD) | 4.41(3.32) | 3.77(2.60) | -0.2432 | 3.87(2.51) | 3.78(2.61) | -0.0348 |
| Median[Q1,Q3] | 3.70[2.00,6.00] | 3.00[2.00,5.00] |  | 3.70[2.10,5.00] | 3.00[2.00,5.00] |  |
| **Tubal factor** | 1783 (65.6%) | 96 (65.8%) | 0.0013 | 90 (62.9%) | 94 (65.7%) | 0.028 |
| **Polycystic ovarian syndrome** | 281 (10.3%) | 40 (27.4%) | 0.1705 | 39 (27.3%) | 39 (27.3%) | 0 |
| **Endometriosis** | 257 (9.5%) | 10 (6.8%) | -0.0261 | 7 (4.9%) | 10 (7.0%) | 0.021 |
| **Basal FSH ,mIU/mL** |  |  |  |  |  |  |
| Mean(SD) | 10.6(105) | 6.66(1.77) | -2.2462 | 6.76(2.48) | 6.62(1.76) | -0.0763 |
| Median[Q1,Q3] | 7.45[6.21,9.40] | 6.28[5.46,7.51] |  | 6.62[5.32,7.60] | 6.20[5.45,7.43] |  |
| **Basal LH ,mIU/mL** |  |  |  |  |  |  |
| Mean(SD) | 5.04(3.39) | 7.28(4.90) | 0.4582 | 8.21(7.61) | 6.94(4.21) | -0.2577 |
| Median[Q1,Q3] | 4.29[3.14,5.82] | 5.99[4.04,8.64] |  | 5.52[3.97,9.41] | 5.89[3.99,8.49] |  |
| **AFC** |  |  |  |  |  |  |
| Mean(SD) | 10.1(6.47) | 15.3(5.78) | 0.9016 | 14.8(6.78) | 15.2(5.75) | 0.075 |
| Median[Q1,Q3] | 8.00[5.00,14.0] | 15.0[10.0,20.0] |  | 15.0[9.00,20.0] | 15.0[10.0,20.0] |  |
| **Agonist protocol** | 1530 (56.3%) | 136 (93.2%) | 0.3684 | 130 (90.9%) | 133 (93.0%) | 0.021 |
| **Antagonist protocol** | 1187 (43.7%) | 10 (6.8%) |  | 13 (9.1%) | 10 (7.0%) |  |
| **Gn starting dose,IU** |  |  |  |  |  |  |
| Mean(SD) | 198(43.3) | 176(41.4) | -0.5186 | 176(45.7) | 176(41.5) | 0 |
| Median[Q1,Q3] | 225[150,225] | 150[150,225] |  | 150[150,225] | 150[150,225] |  |
| **IVF** | 2006 (73.8%) | 103 (70.5%) |  | 105 (73.4%) | 101 (70.6%) |  |
| **ICSI** | 711 (26.2%) | 43 (29.5%) | 0.0328 | 38 (26.6%) | 42 (29.4%) | 0.028 |
| **Whole embryo blastocyst culture** | 545 (20.1%) | 62 (42.5%) | 0.2241 | 59 (41.3%) | 59 (41.3%) | 0 |
| **Freeze-all** | 448 (16.5%) | 62 (42.5%) | 0.2598 | 56 (39.2%) | 59 (41.3%) | 0.021 |
| **Male age,years** |  |  |  |  |  |  |
| Mean(SD) | 34.3(5.42) | 31.9(4.90) | -0.4825 | 32.0(3.99) | 32.0(4.89) | -0.01 |
| Median[Q1,Q3] | 34.0[30.0,38.0] | 31.0[28.0,34.0] |  | 31.0[29.0,35.0] | 31.0[28.5,34.0] |  |
| **Male BMI ,kg/m^2^** |  |  |  |  |  |  |
| Mean(SD) | 24.0(3.45) | 23.8(3.44) | -0.0685 | 23.9(3.55) | 23.8(3.41) | -0.0414 |
| Median[Q1,Q3] | 23.9[21.6,26.0] | 23.7[21.3,26.0] |  | 23.8[21.5,26.0] | 23.7[21.3,26.0] |  |
| **Sperm morphology,%** |  |  |  |  |  |  |
| Mean(SD) | 7.20(4.94) | 7.54(4.66) | 0.0724 | 7.64(8.52) | 7.49(4.61) | -0.031 |
| Median[Q1,Q3] | 6.00[4.00,9.00] | 6.50[4.13,9.48] |  | 6.50[3.95,9.00] | 6.50[4.25,9.45] |  |
| **Total motile sperm count,10^6^/ml** |  |  |  |  |  |  |
| Mean(SD) | 74.5(489) | 51.0(52.1) | -0.4525 | 45.3(43.5) | 50.6(51.6) | 0.1019 |
| Median[Q1,Q3] | 45.1[17.5,89.1] | 38.3[12.4,70.1] |  | 33.9[10.4,69.8] | 38.2[12.5,70.1] |  |

Data were presented as mean±SD and median [first quartile, third quartile] for continuous variables and n (percentage) for categorical variables. *D: Standardized difference. The absolute value of D is less than 0.1, cohorts can be considered to be balanced concerning the demographics being assessed.

TableS6 The Impact of Gn decrease before E_2_ decrease on Pregnancy Outcomes

|  | **Unmatched** | | **P-value** | **Matched** | | **P-value** |
| --- | --- | --- | --- | --- | --- | --- |
|  | N**on decreased** | **Decreased** |  | N**on decreased** | **Decreased** |  |
|  | **(N=2717)** | **(N=146)** |  | **(N=143)** | **(N=143)** |  |
| **Absolute E2 decline, pg/ml** |  |  |  |  |  |  |
| Mean(SD) | -217(394) | -743(1030) | <0.001 | -396(667) | -728(1020) | <0.001 |
| Median[Q1,Q3] | -75.0[-239,-18.0] | -372[-786,-154] |  | -133[-432,-28.0] | -365[-781,-149] |  |
| **Percentage of E2 decline, %** |  |  |  |  |  |  |
| Mean(SD) | 20.2(21.6) | 24.7(24.0) | 0.033 | 22.3(22.2) | 25.2(24.4) | 0.433 |
| Median[Q1,Q3] | 12.6[5.20,27.0] | 13.2[5.41,35.5] |  | 13.5[5.50,31.5] | 13.0[5.58,38.3] |  |
| **Dosage change following the decline, IU** |  |  |  |  |  |  |
| Mean(SD) | 9.57(23.6) | 15.4(30.7) | 0.003 | 9.79(21.7) | 15.7(31.0) | 0.123 |
| Median[Q1,Q3] | 0[0,0] | 0[0,37.5] |  | 0[0,0] | 0[0,37.5] |  |
| **Follicular diameter changes at the decline** |  |  |  |  |  |  |
| Mean(SD) | 1.76(2.11) | 2.66(3.72) | <0.001 | 1.75(3.17) | 2.67(3.76) | 0.005 |
| Median[Q1,Q3] | 1.50[0.500,2.50] | 2.00[1.00,3.50] |  | 1.50[0.800,2.50] | 2.00[1.00,3.50] |  |
| **Dosage used when the decline occurs, IU** |  |  |  |  |  |  |
| Mean(SD) | 2610(946) | 2400(822) | 0.006 | 2770(1000) | 2400(823) | <0.001 |
| Median[Q1,Q3] | 2480[2030,3080] | 2250[1760,2780] |  | 2480[2030,3340] | 2250[1760,2780] |  |
| **Follicle count changes at the decline** |  |  |  |  |  |  |
| Mean(SD) | -0.115(2.55) | -1.01(4.41) | <0.001 | -0.615(4.76) | -0.916(4.38) | 0.125 |
| Median[Q1,Q3] | 0[-1.00,1.00] | -1.00[-3.00,1.00] |  | 0[-2.00,2.00] | -1.00[-3.00,1.00] |  |
| **Dosage adjustment before the decline, IU** |  |  |  |  |  |  |
| Mean(SD) | 2.55(11.8) | -49.1(24.0) | <0.001 | 5.94(16.8) | -49.1(24.1) | <0.001 |
| Median[Q1,Q3] | 0[0,0] | -37.5[-75.0,-37.5] |  | 0[0,0] | -37.5[-75.0,-37.5] |  |
| **Dosage decrease before the decline (%)** | 0 (0%) | 146 (100%) | <0.001 | 0 (0%) | 143 (100%) | <0.001 |
| **Total dosage of Gn, IU** |  |  |  |  |  |  |
| Mean(SD) | 2610(946) | 2400(822) | 0.006 | 2770(1000) | 2400(823) | <0.001 |
| Median[Q1,Q3] | 2480[2030,3080] | 2250[1760,2780] |  | 2480[2030,3340] | 2250[1760,2780] |  |
| **Gn medication duration** |  |  |  |  |  |  |
| Mean(SD) | 11.9(3.78) | 16.7(4.37) | <0.001 | 14.4(4.19) | 16.6(4.39) | <0.001 |
| Median[Q1,Q3] | 11.0[9.00,14.0] | 16.0[14.0,19.0] |  | 14.0[11.0,17.0] | 16.0[14.0,19.0] |  |
| **Estradiol on HCG day, pg/ml** |  |  |  |  |  |  |
| Mean(SD) | 2110(1750) | 4290(2730) | <0.001 | 3410(2600) | 4280(2750) | 0.002 |
| Median[Q1,Q3] | 1550[859,2980] | 4100[2460,5130] |  | 3060[1260,4630] | 4090[2410,5110] |  |
| **LH on HCG day, mIU/mL** |  |  |  |  |  |  |
| Mean(SD) | 1.60(2.45) | 0.796(1.12) | <0.001 | 0.941(1.07) | 0.806(1.13) | 0.078 |
| Median[Q1,Q3] | 0.930[0.490,1.92] | 0.510[0.298,0.860] |  | 0.650[0.330,1.10] | 0.520[0.320,0.860] |  |
| **P on HCG day, ng/ml** |  |  |  |  |  |  |
| Mean(SD) | 0.934(1.20) | 1.10(0.663) | <0.001 | 1.09(0.851) | 1.10(0.663) | 0.438 |
| Median[Q1,Q3] | 0.770[0.510,1.13] | 0.880[0.613,1.41] |  | 0.900[0.575,1.28] | 0.870[0.615,1.41] |  |
| **Oocyte yield** |  |  |  |  |  |  |
| Mean(SD) | 6.81(5.10) | 13.2(7.77) | <0.001 | 10.6(6.81) | 13.1(7.80) | 0.003 |
| Median[Q1,Q3] | 6.00[3.00,9.00] | 12.5[7.25,18.8] |  | 9.00[6.00,15.0] | 12.0[7.00,18.5] |  |
| **Mature oocyte** |  |  |  |  |  |  |
| Mean(SD) | 6.06(4.69) | 11.9(7.48) | <0.001 | 9.49(6.29) | 11.8(7.50) | 0.008 |
| Median[Q1,Q3] | 5.00[3.00,8.00] | 11.0[6.00,16.0] |  | 8.00[5.00,13.0] | 11.0[6.00,16.0] |  |
| **Oocyte maturation rate, %** |  |  |  |  |  |  |
| Mean(SD) | 88.3(19.7) | 89.4(15.5) | 0.056 | 88.7(17.1) | 89.2(15.7) | 0.79 |
| Median[Q1,Q3] | 100[82.4,100] | 93.2[83.6,100] |  | 95.0[83.3,100] | 93.1[83.3,100] |  |
| **Zygote** |  |  |  |  |  |  |
| Mean(SD) | 5.36(4.25) | 10.5(6.80) | <0.001 | 8.29(5.70) | 10.4(6.81) | 0.009 |
| Median[Q1,Q3] | 4.00[2.00,7.00] | 10.0[5.00,14.0] |  | 7.00[4.00,10.5] | 10.0[5.00,14.0] |  |
| **Fertility rate, %** |  |  |  |  |  |  |
| Mean(SD) | 79.4(24.8) | 78.6(21.8) | 0.066 | 78.6(22.3) | 78.6(21.9) | 0.851 |
| Median[Q1,Q3] | 85.7[66.7,100] | 83.6[66.7,93.9] |  | 85.7[66.7,100] | 83.9[66.7,94.4] |  |
| **2 pronuclei embryo** |  |  |  |  |  |  |
| Mean(SD) | 4.28(3.54) | 8.45(5.88) | <0.001 | 6.45(4.60) | 8.36(5.85) | 0.007 |
| Median[Q1,Q3] | 3.00[2.00,6.00] | 8.00[4.00,11.0] |  | 6.00[3.00,9.00] | 8.00[4.00,11.0] |  |
| **Cleavage** |  |  |  |  |  |  |
| Mean(SD) | 4.57(3.74) | 8.92(5.94) | <0.001 | 7.03(4.98) | 8.81(5.91) | 0.009 |
| Median[Q1,Q3] | 4.00[2.00,6.00] | 8.00[4.00,13.0] |  | 6.00[3.00,9.00] | 8.00[4.00,13.0] |  |
| **Cleavage 2 pronuclei embryo** |  |  |  |  |  |  |
| Mean(SD) | 4.19(3.49) | 8.31(5.77) | <0.001 | 6.33(4.55) | 8.22(5.73) | 0.007 |
| Median[Q1,Q3] | 3.00[2.00,6.00] | 8.00[4.00,11.0] |  | 5.00[3.00,9.00] | 8.00[3.50,11.0] |  |
| **No embryo** | 140 (5.2%) | 6 (4.1%) | 0.715 | 5 (3.5%) | 6 (4.2%) | ＞0.999 |
| **Good quality embryos** |  |  |  |  |  |  |
| Mean(SD) | 2.58(2.67) | 5.40(4.32) | <0.001 | 4.12(3.46) | 5.34(4.31) | 0.023 |
| Median[Q1,Q3] | 2.00[1.00,4.00] | 5.00[2.00,8.00] |  | 3.00[2.00,6.00] | 5.00[2.00,8.00] |  |
| **Good quality embryo rate, %** |  |  |  |  |  |  |
| Mean(SD) | 50.4(35.9) | 54.9(28.9) | 0.223 | 56.1(31.4) | 54.9(29.2) | 0.648 |
| Median[Q1,Q3] | 50.0[16.7,80.0] | 60.6[34.4,76.9] |  | 60.0[37.5,77.8] | 60.0[33.3,77.4] |  |
| **Cumulative live birth rate, %** | 1460 (53.7%) | 116 (79.5%) | <0.001 | 98 (68.5%) | 114 (79.7%) | 0.043 |
| **Fresh embryo transfer,N** | **(N=1973)** | **(N=234)** |  | **(N=82)** | **(N=78)** |  |
| **Fresh embryo transfer number** |  |  |  |  |  |  |
| 1 | 814 (41.3%) | 98 (41.9%) | 0.906 | 33 (40.2%) | 25 (32.1%) | 0.186 |
| 2 | 1117 (56.6%) | 132 (56.4%) |  | 47 (57.3%) | 53 (67.9%) |  |
| 3 | 42 (2.1%) | 4 (1.7%) |  | 2 (2.4%) | 0 (0%) |  |
| **Fresh blastocyst transfer** | 215 (10.9%) | 18 (7.7%) | 0.163 | 11 (13.4%) | 13 (16.7%) | 0.723 |
| **Fresh cycle live birth** | 939 (47.6%) | 102 (43.6%) | 0.276 | 42 (51.2%) | 52 (66.7%) | 0.068 |
| **aOR (95% CI)*** | ref | 1.21(0.73,2.01) | 0.461 | ref | 0.71(0.3,1.66) | 0.43 |

All models were adjusted for female and male age, BMI, history of live birth, duration of infertility, tubal factor, PCOS, endometriosis, basic FSH, LH, antral follicle, agonist protocol, Antagonist protocol, Gn start dose, IVF, ICSI, whole embryo blastocyst culture, freeze-all, Sperm normal morphology, total mobil sperm count. The cumulative live birth rate was the dependent variable.

Table S8 E-values of the association between E_2_ decline and cumulative live birth rate.

| **Type** | **OR (95% CI)** | **RR (95% CI)** | **E-values (95% CI)** |
| --- | --- | --- | --- |
| **Overall E2 decline versus control** | 0.83(0.76,0.91) | 0.91(0.87,0.95) | 1.43(1.27,1.56) |
| **Female age, per year increase** | 0.92(0.91,0.93) | 0.96(0.95,0.96) | 1.25(1.23,1.27) |
| **Without a consecutive estradiol decline at two visits versus control** | 0.87(0.79,0.96) | 0.93(0.89,0.98) | 1.35(1.17,1.5) |
| **With a consecutive estradiol decline at two visits versus control** | 0.58(0.45,0.74) | 0.76(0.67,0.86) | 1.95(1.6,2.35) |
| **Without previous dosage adjustment before estradiol decline** | 0.87(0.79,0.95) | 0.93(0.89,0.97) | 1.35(1.19,1.5) |
| **With previous dosage adjustment before estradiol decline** | 0.69(0.56,0.84) | 0.83(0.75,0.92) | 1.7(1.41,2.01) |

All models were adjusted for female and male age, BMI, history of live birth, duration of infertility, tubal factor, PCOS, endometriosis, basic FSH, LH, antral follicle, agonist protocol, Antagonist protocol, Gn start dose, IVF, ICSI, whole embryo blastocyst culture, freeze-all, Sperm normal morphology, total mobil sperm count. The cumulative live birth rate was the dependent variable.
